# Supplementary material for: Identification of Freezing-Responsive microRNAs and Their Targets in Chinese Jujube by Small RNA and Degradome Sequencing
Source: Int J Mol Sci. 2026 Sep 4;27(17):7912. doi: 10.3390/ijms27177912 (PMC13566890; doi:10.3390/ijms27177912)
Supplement: Supplementary file 1 [file ijms-27-07912-s001.zip › Supplementary Material S2/novel_miR_166.pdf]

Provisional ID	:	novel_miR_166
Score total	:	99615.5
Score for star read(s)	:	3.9
Score for read counts	:	99609.2
Score for mfe	:	0.7
Score for randfold	:	1.6
Score for cons. seed	:	
Total read count	:	195391
Mature read count	:	191629
Loop read count	:	16
Star read count	:	3746

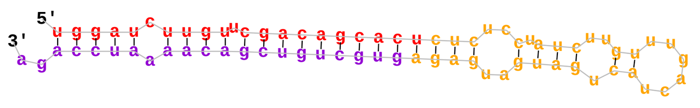

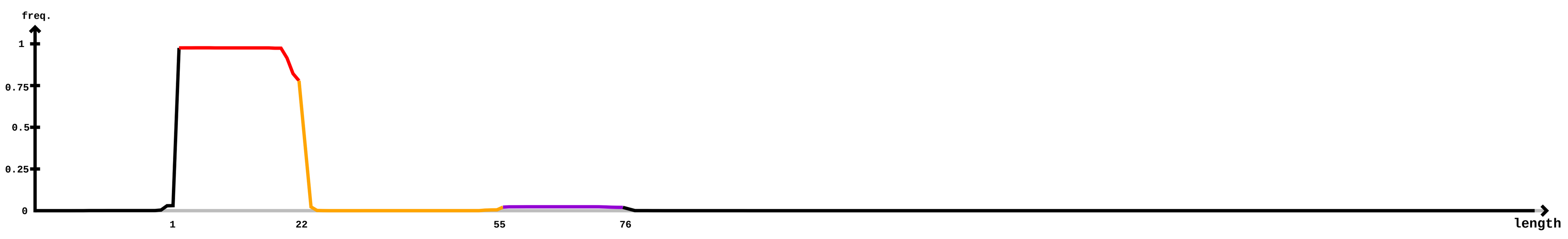

Mature Star

```
5'- uccaucuuguucguuuacccucuggaucuuguucgacagcacucucuccuaucuuguuugacuacugaugaugagagugcugucgacaaaauccagaaggugcugucgaauaagauacagauacagaggaacgagcaacuguaccuacugucaugacaaucuucuucauauucucuucaauuuuuauuuauuuuuuuucuuauuguucguguuuuauuuuuuuuuuuuaauuuccagauuagauuuaccu -3'  obs
    uccaucuuguucguuuacccucuggaucuuguucgacagcacucucuccuaucuuguuugacuacugaugaugagagugcugucgacaaaauccagaaggugcugucgaauaagauacagauacagaggaacgagcaacuguaccuacugucaugacaaucuucuucauauucucuucaauuuuuauuuauuuuuuuucuuauuguucguguuuuauuuuuuuuuuuuaauuuccagauuagauuuaccu       exp
    ...((((((((((..((((.(((((((.((((.((((((((((((((..(.(((..((......)).))))..)))))))))))))))))).))))))).))))....))))))))))......((((((.(((......))).))).))))(((((((((.......................................))))).)))))............((((((((....))))))))....... reads  mm  sample
    .ccaucuuguucguuuacc........                  1   0   LT-1
    .ccaucuuguucguuuacccucugg........            5   0   LCK-3
    .ccaucuuguucguuuacccucuggauc........         1   0   LT-3
    ...aucuuguucguuuacccucugga........           2   0   JCK-1
    .....cuuguucguuuacccucuggauc........         4   0   LT-2
    ......uuguucguuuaccUucuggauc........         2   1   JCK-1
    ......uuguucguuuaccUucuggauc........         3   1   JCK-3
    ......uuguucguuuaccUucuggauc........         6   1   JCK-2
    ......uuguucguuuaccUucuggauc........         2   1   LT-2
    .......uguucguuuacccucuggauc........         1   0   JCK-2
    .......uguucguuuaccUucuggauc........         12  1   LT-3
    .......uguucguuuaccUucuggauc........         30  1   JCK-3
    .......uguucguuuaccUucuggauc........         10  1   JCK-1
    .......uguucguuuacccucuggauc........         2   0   JCK-3
    .......uguucguuuaccUucuggauc........         12  1   LT-1
    .......uguucguuuacccucuggauc........         1   0   JCK-1
    .......uguucguuuaccUucuggauc........         31  1   JCK-2
    .......uguucguuuaccUucuggauc........         3   1   LT-2
    ..........ucguuuacccucuggaucuug........      1   0   LT-2
    .............uuuacccucuggaucuuguA........    1   1   JCK-1
    .............uuuacccucuggaucuuguuc........   1   0   JCK-2
    .............uuuacccucuggaucuuguuc........   1   0   JCK-3
    ..................ccucuggaucuuguucgaca........      1  0  JCK-1
    ..................ccucuggaucuuguucgacag........     5  0  LT-3
    ..................ccucuggaucuuguucgacagc........    2  0  LT-1
    ..................ccucuggaucuuguucgacagc........    4  0  JCK-3
    ..................ccucuggaucuuguucgacagc........    2  0  LT-2
    ..................ccucuggaucuuguucgacagc........    8  0  JCK-2
    ..................ccucuggaucuuguucgacagc........    2  0  LT-3
    ..................ccucuggaucuuguucgacagc........    6  0  JCK-1
    ..................ccucuggaucuuguucgacagcacu........ 1  0  JCK-2
    ..................ccucuggCucuuguucgacagcacu........ 1  1  JCK-2
    ..................ccucuggaucuuguucgacagcacu........ 1  0  JCK-1
```

Mature **Star**

uccaucuuguucguuuacccucuggaucuuguucgacagcacucucuccuaucuuguuuugacuacugaugaugagagugcugucgacaaaauccagaaggugcugucgaauaagauacagauacagaggaacgagcaacuguaccuacugucaugacaaucuucuucauauucucuucaauuuuuauuuauuuuuuuucuuauuguucguguuuuauuuuuuuuuuuaauuuccagauuagauuuaccu			
.................ccucuggaucuuguucgacagcacu...	1	0	JCK-3
..................cucuggaucuuguucgac...	1	0	LT-3
..................cucuggaucuuguucgaca...	2	0	LT-1
..................cucuggaucuuguucgaca...	6	0	LT-3
..................cucuggaucuuguucgaca...	8	0	LCK-3
..................cucuggaucuuguucgaca...	6	0	LT-2
..................cucuggaucuuguucgacagc...	43	0	JCK-2
..................cucuggaucuuguucgacagc...	34	0	JCK-3
..................cucuggaucuuguucgacagc...	5	0	LCK-3
..................cucuggCucuuguucgacagc...	1	1	JCK-2
..................cucuggaucuuguucgacagc...	8	0	LT-2
..................cucuggaucuuguucgacagc...	20	0	LT-1
..................cCcuggaucuuguucgacagc...	2	1	LT-1
..................cucuggaucuuguucgacagc...	22	0	LT-3
..................cucuggaucuuguucgacagc...	36	0	JCK-1
..................cucuggaucuuguucgacCgca...	1	1	JCK-1
..................cucuggaucuuguucgacagca...	12	0	LT-3
..................cucuggaucuuguucgacagca...	137	0	JCK-2
..................cucuggaucuuguucgacagca...	103	0	JCK-3
..................cucuggaucuuguucgCcagca...	1	1	LT-1
..................cucuggaucuuNuucgacagca...	1	1	JCK-1
..................cucuggaucuuguucgacagca...	81	0	JCK-1
..................cucuggaucuuguucgacagca...	24	0	LCK-3
..................cucuggaucuuguucgacagca...	19	0	LT-1
..................cucuggaucuuguucgacagcU...	3	1	JCK-2
..................Aucuggaucuuguucgacagca...	2	1	JCK-3
..................cucuggaucuuguucgacagcU...	1	1	JCK-1
..................cucuggaucuuguucgacagUa...	2	1	JCK-2
..................cucuggaucuuguucgacagcG...	1	1	JCK-1
..................cNcuggaucuuguucgacagca...	1	1	JCK-3
..................cucuggaucuuguucgacaNca...	1	1	JCK-1
..................cucuggaucuuguucgacagca...	6	0	LT-2
..................cNcuggaucuuguucgacagca...	1	1	JCK-2
..................cucuggaucuuguucgaAagca...	1	1	JCK-1
..................cuUuggaucuuguucgacagca...	1	1	JCK-3
..................cucuggaucuuguucgacagcacu...	1	0	JCK-3
..................cucuggaucuuguucgacagcacucu...	1	0	LT-3
...................ucuggaucuuguucgaca...	17	0	JT-3
...................ucuggaucuuguucgCca...	1	1	LT-3
...................ucuggaucuuguucgacU...	2	1	LT-1
...................ucuggaucuuguucgaca...	12	0	JT-1
...................ucuggaucuuguucgacU...	1	1	JCK-1
...................ucuggaucuuguucgaca...	53	0	LT-3
...................ucuggaucuuguucgaca...	23	0	JCK-2
...................ucuggaucuuguucgacU...	2	1	LCK-3
...................ucuggaucuuguucgaca...	37	0	LT-2
...................ucuggaucuuguucgacU...	1	1	LT-3
...................ucuggaucuuguucgaca...	76	0	LCK-3
...................ucuggaucuuguucgaca...	14	0	JT-2
...................ucuggaucuuguucgaca...	17	0	LT-1
...................ucuggaucuuguucgacU...	1	1	JCK-2
...................ucuggaucuuguucgaca...	25	0	JCK-1
...................ucuggaucuuguucgaca...	22	0	JCK-3
...................ucuggaucuuguucgacaA...	1	1	LT-1
...................ucuggaucuuguucgacag...	1	0	JCK-1
...................ucuggaucuuguucgacaU...	2	1	JCK-2
...................ucuggaucuuguucgacaU...	2	1	JCK-3
...................ucuggaucuuguucgacag...	1	0	LT-3
...................ucuggaucuuguucgacagc...	33	0	JCK-1
...................ucuggaucuuguucgacagc...	4	0	JT-3
...................ucuggaucuuguucgacagc...	80	0	LT-3
...................ucuggaucuuguucgacagU...	1	1	LT-1
...................ucuggaucuuguucgacagc...	20	0	LCK-1
...................ucuggaucuuguucgacagc...	28	0	LCK-2
...................ucuggaucuuguucgacagc...	56	0	JCK-2
...................ucuggaucuuguucgacagc...	69	0	LT-2
...................ucuggCucuuguucgacagc...	1	1	LT-1
...................ucuggaucuuguucgacagc...	5	0	JT-2
...................ucuggaucuuguucgacagc...	62	0	LCK-3
...................ucuggaucuuguucgacagc...	52	0	LT-1

Mature **Star**

uccaucuuguucguuuacccucuggaucuuguucgacagcacucucuccuaucuuguuugacuacugaugaugagagugcugucgacaaaauccagaaggugcugucgaauaagauacagauacagaggaacgagcaacuguaccuacugucaugacaaucuucuucauauucucuucaauuuuuauuuauuuuuuuucuuauuguucguguuuuauuuuuuuuuuuaauuuccagauuagauuuaccu

....................ucuggaucuuguucgacagc	29	0	JCK-3
....................ucuggNucuuguucgacagc	1	1	LCK-1
....................ucuggaucuuguucgacagca	882	0	JCK-1
....................ucuggaucuuguucgCcagca	1	1	JCK-1
....................ucuggaucuugNucgacagca	2	1	JCK-1
....................ucuggaucCuguucgacagca	1	1	LT-2
....................ucuggaucuuguucgacagca	76	0	LCK-3
....................ucuggaucuuguucgacagca	56	0	JT-1
....................ucuggaucuuguucgacagca	83	0	LT-2
....................ucuggaucuuguucgacagca	128	0	LT-3
....................ucuggaucuuguucgacaUca	1	1	JCK-1
....................ucuggaucuuguucgacagcU	3	1	JCK-3
....................ucuggaucuuguucgacagca	31	0	LCK-2
....................uUuggaucuuguucgacagca	5	1	JCK-2
....................ucuggaucuuguucgaAagca	1	1	JCK-1
....................uNuggaucuuguucgacagca	2	1	JCK-2
....................ucuggaucuuguucgacagca	1162	0	JCK-3
....................ucuggaucuuguucgaAagca	1	1	JCK-3
....................ucCggaucuuguucgacagca	1	1	JCK-3
....................Ccuggaucuuguucgacagca	1	1	JCK-2
....................ucuggaucuuguucgacagcU	2	1	JCK-1
....................ucuggaucCuguucgacagca	1	1	JCK-2
....................ucuggaucuuguucgacagca	109	0	LT-1
....................ucuggaucuuguucgacagcU	8	1	JCK-2
....................uUuggaucuuguucgacagca	5	1	JCK-3
....................ucuggaucuuguucAacagca	1	1	JCK-1
....................ucuggaucuuguucgacagca	27	0	LCK-1
....................ucNggaucuuguucgacagca	1	1	LCK-1
....................ucuggaucuuguucgCcagca	1	1	LT-1
....................ucuggaucuuguucgCcagca	1	1	JT-1
....................ucuggaucuuguucgacagca	1463	0	JCK-2
....................uUuggaucuuguucgacagca	5	1	JCK-1
....................ucuggaucuuguucgacagcU	3	1	LT-2
....................ucuggCucuuguucgacagca	4	1	JCK-2
....................ucuggaucuuguucgacaAca	1	1	JCK-2
....................Ccuggaucuuguucgacagca	1	1	LT-3
....................ucuggaucuuguucgaAagca	1	1	JCK-2
....................ucuggaucuuguucgacagcN	1	1	JCK-1
....................ucuggaucuuguucgacagcU	3	1	LCK-3
....................ucuggaucuuguucgacagca	9	0	JT-2
....................ucuggaucuuguucgacagcC	1	1	JCK-1
....................ucuggauAuuguucgacagca	1	1	JCK-3
....................ucuggaucuuguucgaUagca	1	1	JCK-3
....................ucuggaucuuguucgacagca	19	0	JT-3
....................ucuggCucuuguucgacagca	1	1	JCK-3
....................ucugAaucuuguucgacagca	1	1	JCK-1
....................ucuggaucuuguucgacaUca	3	1	JCK-2
....................ucuggaucuuguucgacagcU	2	1	LT-3
....................ucuggaucuuguucgacagUa	1	1	JCK-3
....................ucuggaucuuguucgacagcac	7	0	LT-2
....................ucuggaucuuguucgacagcac	10	0	LT-1
....................ucuggaucuuguucgacagcac	16	0	JCK-1
....................ucuggaucuuguucgacagcaA	1	1	JCK-3
....................ucuggaucuuguucgacagcac	37	0	JCK-2
....................ucuggaucuuguucgacagcac	16	0	LCK-3
....................ucuggaucuuguucgacagcac	30	0	JCK-3
....................ucuggaucuuguucgacagcac	11	0	LT-3
....................ucuggaucuuguucgacagcacA	2	1	JCK-1
....................ucuggaucuuguucgacagcacu	4	0	JCK-1
....................ucuggaucuuguucgacagcacu	5	0	JCK-3
....................ucuggaucuuguucgacagcacu	3	0	LT-3
....................ucuggaucuuguucgacagcacu	8	0	LCK-3
....................ucuggaucuuguucgacagcaUu	1	1	LT-3
....................ucuggaucuuguucgacagcacC	1	1	LT-3
....................ucuggaucuuguucgacagcacA	1	1	JCK-2
....................ucuggaucuuguucgacagcacu	2	0	LT-2
....................ucuggaucuuguucgacagcacA	1	1	JCK-3
....................ucuggaucuuguucgacagcacu	9	0	JCK-2
....................ucuggaucuuguucgacagcaAu	3	1	LT-3
....................ucuggaucuuguucgacagcacuU	1	1	LT-1

```
                    Mature                                           Star
uccaucuuguucguuuacccucuggaucuuguucgacagcacucucuccuaucuuguuugacuacugaugaugagagugcugucgacaaaauccagaaggugcugucgaauaagauacagauacagaggaacgagcaacuguaccuacugucaugacaaucuucuucauauucucuucaauuuuuauuuauuuuuuuucuuauuguucguguuuuauuuuuuuuuuuuaauuuccagauuagauuuaccu
....................ucuggaucuuguucgacagcacuc..............................................................................................................................................................................................   3     0    JCK-2
....................ucuggaucuuguucgacagcacuc..............................................................................................................................................................................................   1     0    LT-3
....................ucuggaucuuguucgacagcacuc..............................................................................................................................................................................................   5     0    JCK-1
....................ucuggaucuuguucgacagcacuc..............................................................................................................................................................................................   3     0    LT-1
....................ucuggaucuuguucgacagcacuc..............................................................................................................................................................................................   1     0    JCK-3
....................ucuggaucuuguucgacagcacucu.............................................................................................................................................................................................   1     0    LT-1
....................ucuggaucuuguucgacagcacucu.............................................................................................................................................................................................   2     0    JCK-2
.....................cuggaucuuguucgacagc..................................................................................................................................................................................................   1     0    LCK-3
.....................cuggaucuuguucgacagc..................................................................................................................................................................................................   3     0    LT-2
.....................cuggaucuuguucgacagc..................................................................................................................................................................................................   4     0    LT-3
.....................cuggaucuuguucgacagc..................................................................................................................................................................................................   1     0    LT-1
.....................cuggaucuuguucgacagca.................................................................................................................................................................................................   4     0    LT-3
.....................cuggaucuuguucgacagca.................................................................................................................................................................................................   15    0    JCK-1
.....................cuggaucuuguucgacagca.................................................................................................................................................................................................   2     0    LT-1
.....................cuggaucAuguucgacagca.................................................................................................................................................................................................   1     1    LT-1
.....................cuggaucuuguucgacagca.................................................................................................................................................................................................   7     0    LCK-3
.....................cuggaucuuguucgacagca.................................................................................................................................................................................................   12    0    JCK-3
.....................cuggaucuuguucgacagca.................................................................................................................................................................................................   17    0    JCK-2
.....................cuggaucuuguucgacagcU.................................................................................................................................................................................................   1     1    JCK-3
.....................cuggaucuuguucgacagcac................................................................................................................................................................................................   4     0    LT-2
.....................cuggaucuuguucgacagcac................................................................................................................................................................................................   2     0    JCK-2
.....................cuggaucuuguucgacagcac................................................................................................................................................................................................   1     0    JCK-3
.....................cuggaucuuguucgacagcacu...............................................................................................................................................................................................   2     0    LT-3
.....................cuggaucuuguucgacagcacu...............................................................................................................................................................................................   27    0    JCK-2
.....................cuggaucuuguucgacagcacu...............................................................................................................................................................................................   3     0    LT-2
.....................cuggaucuuguucgacagcacu...............................................................................................................................................................................................   13    0    JCK-3
.....................cuggaucuuguucgacagcacu...............................................................................................................................................................................................   2     0    LT-1
.....................cuggaucuuguucgacagcacu...............................................................................................................................................................................................   15    0    JCK-1
.....................cuggaucuuguucgacagcacu...............................................................................................................................................................................................   5     0    LCK-3
.....................Uuggaucuuguucgacagcacu...............................................................................................................................................................................................   1     1    JCK-3
.....................cuggaucuuguucgacagcacuc..............................................................................................................................................................................................   6     0    JCK-2
.....................cuggaucuuguucgacagcacuc..............................................................................................................................................................................................   1     0    JCK-1
.....................cuggaucuuguucgacagcacuc..............................................................................................................................................................................................   2     0    LT-3
.....................cuggaucuuguucgacagcacuc..............................................................................................................................................................................................   1     0    JCK-3
.....................Uuggaucuuguucgacagcacuc..............................................................................................................................................................................................   1     1    JCK-2
......................uggaucuuguucgacaAc..................................................................................................................................................................................................   1     1    LT-2
......................uNgaucuuguucgacagc..................................................................................................................................................................................................   1     1    LCK-1
......................uggaucuuguucgacagc..................................................................................................................................................................................................   454   0    JCK-1
......................uggCucuuguucgacagc..................................................................................................................................................................................................   3     1    JCK-3
......................ugNaucuuguucgacagc..................................................................................................................................................................................................   1     1    LCK-1
......................uNgaucuuguucgacagc..................................................................................................................................................................................................   1     1    JCK-2
......................uNgaucuuguucgacagc..................................................................................................................................................................................................   1     1    LT-3
......................uggaucuuguucgacagU..................................................................................................................................................................................................   5     1    LCK-3
......................uggaucuuguucgacagc..................................................................................................................................................................................................   48    0    JT-2
......................uggaucuuguucgCcagc..................................................................................................................................................................................................   2     1    LCK-2
......................uggaucuuguucgacagc..................................................................................................................................................................................................   81    0    JT-1
......................uggaucuuguucgacagU..................................................................................................................................................................................................   1     1    JCK-1
......................ugAaucuuguucgacagc..................................................................................................................................................................................................   1     1    JCK-3
......................uggaucuuguucgacagc..................................................................................................................................................................................................   616   0    JCK-3
......................uggaucuuguucgacCgc..................................................................................................................................................................................................   1     1    LT-2
......................uAgaucuuguucgacagc..................................................................................................................................................................................................   1     1    LT-2
......................uggaucuuguucgaAagc..................................................................................................................................................................................................   1     1    LCK-3
......................uggCucuuguucgacagc..................................................................................................................................................................................................   1     1    LCK-1
......................ugUaucuuguucgacagc..................................................................................................................................................................................................   1     1    LT-3
......................uggaucuuguucgacagc..................................................................................................................................................................................................   890   0    LT-1
......................uggaucuuguucgNcagc..................................................................................................................................................................................................   2     1    LCK-1
......................uggaucuuguucgacagc..................................................................................................................................................................................................   2025  0    LT-2
......................uggaucuuguuNgacagc..................................................................................................................................................................................................   1     1    JCK-2
......................uggaucuuguucgacagc..................................................................................................................................................................................................   3342  0    LCK-3
......................uggCucuuguucgacagc..................................................................................................................................................................................................   3     1    LT-1
......................ugAaucuuguucgacagc..................................................................................................................................................................................................   1     1    LCK-3
......................uggaucuuguucgaUagc..................................................................................................................................................................................................   1     1    LCK-3
......................uUgaucuuguucgacagc..................................................................................................................................................................................................   1     1    LT-3
......................uggaucuuguucgCcagc..................................................................................................................................................................................................   3     1    JCK-2
......................uggaucuuguucgacagA..................................................................................................................................................................................................   1     1    LT-3
......................uggaucuuguucgCcagc..................................................................................................................................................................................................   1     1    LT-3
......................ugNaucuuguucgacagc..................................................................................................................................................................................................   1     1    LCK-3
......................uggaucuuguucgCcagc..................................................................................................................................................................................................   4     1    LCK-3
......................uggaucuuguucgacagU..................................................................................................................................................................................................   3     1    JCK-3
......................uggaucuuguucgCcagc..................................................................................................................................................................................................   8     1    LT-2
```

Mature Star

uccaucuuguucguuuacccucuggaucuuguucgacagcacucucuccuaucuuguuugacuacugaugaugagagugcugucgacaaaauccagaaggugcugucgaauaagauacagauacagaggaacgagcaacuguaccuacugucaugacaaucuucuucauauucucuucaauuuuuauuuauuuuuuuucuuauuguucguguuuuauuuuuuuuuuuaauuuccagauuagauuuaccu			
......................ugNaucuuguucgacagc..........	4	1	LT-2
......................uggGucuuguucgacagc..........	1	1	JT-1
......................uAgaucuuguucgacagc..........	1	1	LCK-1
......................uggaucuuguucgacagc..........	659	0	LCK-1
......................uggaucuuguucgacagc..........	794	0	JCK-2
......................ugAaucuuguucgacagc..........	1	1	LCK-1
......................uggaucuuguucgaUagc..........	1	1	LT-2
......................ugAaucuuguucgacagc..........	2	1	LT-2
......................uAgaucuuguucgacagc..........	2	1	LCK-3
......................uggaucuuguucgacGgc..........	1	1	JCK-2
......................uggGucuuguucgacagc..........	2	1	JCK-1
......................ugAaucuuguucgacagc..........	2	1	JCK-2
......................uggaucuuguucgacagU..........	1	1	LT-2
......................uggaucuuguucAacagc..........	1	1	LT-2
......................uggaucuuguucgCcagc..........	1	1	LCK-1
......................uggaucuuguucgacaUc..........	6	1	LT-2
......................uggaucuuguucgCcagc..........	2	1	LT-1
......................uggaucuuguucgacaAc..........	1	1	LCK-3
......................uggaCcuuguucgacagc..........	4	1	LCK-3
......................uggaucuuguucgaAagc..........	1	1	LT-2
......................uggaucuuguucgacaUc..........	2	1	JCK-3
......................uggaucuuguucgacagc..........	74	0	JT-3
......................uggaucuuguucgacagc..........	1173	0	LT-3
......................uggCucuuguucgacagc..........	3	1	LT-3
......................uggaucuuguucgacagU..........	15	1	LT-3
......................uggCucuuguucgacagc..........	6	1	LCK-3
......................uggaucuuguucgacagU..........	3	1	LT-1
......................uggaucuuguucUacagc..........	1	1	JCK-3
......................uggCucuuguucgacagc..........	19	1	LT-2
......................Cggaucuuguucgacagc..........	1	1	LT-2
......................uggaucuuguuNgacagc..........	1	1	JCK-1
......................uggaucuuguuNgacagc..........	1	1	JT-1
......................uggCucuuguucgacagc..........	1	1	JCK-1
......................uggCucuuguucgacagc..........	1	1	LCK-2
......................uAgaucuuguucgacagc..........	2	1	JCK-2
......................uggaucuuguucgacaUc..........	1	1	JCK-1
......................uNgaucuuguucgacagc..........	1	1	JT-1
......................uggaucuuguucgacagc..........	663	0	LCK-2
......................uggaucuuguucgacagU..........	2	1	JCK-2
......................uggaucuuguucgGcagc..........	1	1	LCK-3
......................uggaucuuguucgaUagc..........	1	1	JCK-1
......................uggaucuuguucgacaAc..........	1	1	LT-1
......................uggaucuuguucgacCgc..........	1	1	JCK-3
......................uggaucuuguucgCcagc..........	2	1	JCK-3
......................uggaucuuguucgacCgca..........	2	1	LCK-2
......................uggaucuuguucgCcagca..........	3	1	LT-2
......................uggaucuuguucgacagUa..........	10	1	JT-1
......................uAgaucuuguucgacagca..........	2	1	LCK-3
......................uggaucuuguucgCcagca..........	1	1	LCK-3
......................uggCucuuguucgacagca..........	1	1	JCK-3
......................uggaucuuguucgCcagca..........	2	1	JCK-3
......................uggaucuuguuGgacagca..........	9	1	LCK-3
......................uggaucuuguucgacagcG..........	2	1	LT-3
......................uggaucuuguucgacagca..........	1963	0	LCK-2
......................uggaucuuguucgacagcU..........	211	1	LT-2
......................uggaucCuguucgacagca..........	1	1	LCK-1
......................uggaucuuguucgacagca..........	575	0	JT-1
......................uggaucuuguucgacagca..........	496	0	LT-1
......................uNgaucuuguucgacagca..........	2	1	JCK-3
......................uggaucuuguucgacagcC..........	1	1	LCK-2
......................uggGucuuguucgacagca..........	1	1	JT-2
......................uggaucuuguucgacagUa..........	4	1	JCK-3
......................uAgaucuuguucgacagca..........	1	1	JCK-2
......................uggaucuuguucgaGagca..........	1	1	LCK-1
......................uggCucuuguucgacagca..........	5	1	LT-1
......................uggCucuuguucgacagca..........	1	1	JCK-2
......................uggaucuuguucgaUagca..........	1	1	LT-2
......................uggaucuuguucgCcagca..........	1	1	JT-1
......................uggCucuuguucgacagca..........	5	1	LT-2
......................uggaucuuguucgacaAca..........	1	1	LT-1

Mature Star

uccaucuuguucguuuacccucuggaucuuguucgacagcacucucuccuaucuuguuugacuacugaugaugagagugcugucgacaaaauccagaaggugcugucgaauaagauacagauacagaggaacgagcaacuguaccuacugucaugacaaucuucuucauauucucuucaauuuuuauuuauuuuuuuucuuauuguucguguuuuauuuuuuuuuuuaauuuccagauuagauuuaccu

uggCucuuguucgacagca	2	1	LCK-1
uggaucuuguucgaNagca	4	1	LCK-1
uggaucuuguuNgacagca	5	1	JCK-1
uggaucuuguucgaAagca	3	1	LCK-2
uggauUuuguucgacagca	1	1	LCK-3
uggaucuuguucAacagca	1	1	LT-3
uggaucCuguucgacagca	1	1	JT-1
uNgaucuuguucgacagca	3	1	JCK-2
uUgaucuuguucgacagca	1	1	LCK-2
uggaucuuguucgacCgca	1	1	LT-3
uggaucuuguucgCcagca	1	1	LCK-2
uggaucuuguucgacagca	529	0	JCK-1
uggaucuuguucgacCgca	2	1	JCK-2
uggGucuuguucgacagca	1	1	JT-1
uggaucuuAuucgacagca	1	1	LCK-1
ugCaucuuguucgacagca	1	1	LT-2
uggaucuuguucgacagca	2250	0	LCK-3
uggaucuuguucgacagcU	68	1	LCK-1
uggaucuuguucgacagAa	1	1	LT-3
uggCucuuguucgacagca	2	1	LCK-3
uggaucuuguucgacaUca	3	1	JCK-2
uggaucuuguucgNcagca	6	1	LCK-1
uggaucuuguucgacagcU	56	1	JT-1
uggaucCuguucgacagca	1	1	JCK-1
uAgaucuuguucgacagca	1	1	LT-2
uggaucCuguucgacagca	1	1	LT-3
uggaucuuguucgacagcU	9	1	JT-2
uggaucuuguucgacCgca	1	1	LCK-3
uggauNuuguucgacagca	3	1	LCK-1
uggaucuuguucgacagcU	75	1	LT-1
Cggaucuuguucgacagca	1	1	LCK-2
uNgaucuuguucgacagca	1	1	LCK-1
uggaucuuguucgacagcU	155	1	JCK-1
uggaucuuguucgacagUa	1	1	JCK-1
uggaucuuguucgaUagca	4	1	JCK-1
Cggaucuuguucgacagca	1	1	LT-2
uggaucuuguucgacCgca	2	1	LT-2
ugAaucuuguucgacagca	1	1	LT-2
ugNaucuuguucgacagca	5	1	LT-2
uggaucuuguucgacaAca	1	1	JT-1
uggaucuuguucgacagGa	1	1	JCK-2
uggaucuuguucgaAagca	1	1	LT-3
uggaucuuguucgaUagca	1	1	JCK-3
uggGucuuguucgacagca	1	1	LCK-2
uggaucuuguucgacagcG	3	1	JCK-2
uggaucuuguucgacagcC	1	1	JCK-2
uNgaucuuguucgacagca	3	1	JT-1
uggaucuuguucgacagca	1113	0	LT-3
uggaucuuguucgaAagca	1	1	LT-2
uggaucuuguucgacagGa	1	1	JCK-1
uggaucuuguucgacaUca	1	1	LT-3
uggaucuuguucgacagUa	5	1	LT-3
uggCucuuguucgacagca	8	1	LCK-2
uggaucuuguucgacagcU	5	1	JT-3
uggaucuuguucgacagcC	1	1	LCK-3
uggaucuuguucgacagcC	1	1	JT-1
uggaucuuguucgacagca	1924	0	LCK-1
uggaucuuguucgacagUa	11	1	LT-2
uggCucuuguucgacagca	1	1	LT-3
uggaucuuguucgacagcU	194	1	JCK-2
uggaucuuguuNgacagca	2	1	JT-1
uggaucuuguucgacagca	606	0	JCK-3
uUgaucuuguucgacagca	1	1	LT-3
uggaucuuguucgacagcC	2	1	LT-3
uggaucuuguucgacaUca	15	1	LCK-3
uggaucuuguucgaUagca	4	1	JCK-2
uggaucuuguucgacagcU	62	1	LCK-2
uggNucuuguucgacagca	1	1	JCK-1
uggaucuuguucgaUagca	2	1	LCK-2
uggaucuuguuNgacagca	1	1	JT-2

Mature **Star**

uccaucuuguucguuuacccucuggaucuuguucgacagcacucucuccuaucuuguuugacuacugaugaugagagugcugucgacaaaauccagaaggugcugucgaauaagauacagauacagaggaacgagcaacuguaccuacugucaugacaaucuucuucauauucucuucaauuuuuauuuauuuuuuuucuuauuguucguguuuuauuuuuuuuuuuuaauuuccagauuagauuuaccu

......................uggGucuuguucgacagca	1	1	LCK-3
......................ugAaucuuguucgacagca	1	1	JCK-2
......................uggaCcuuguucgacagca	2	1	JCK-3
......................uggaucuuguucgacagcU	162	1	JCK-3
......................uggaucuuguucgacaAca	1	1	LCK-1
......................uggaucuuguucgacaUca	1	1	JCK-3
......................uggaucuuguucgacagcU	299	1	LCK-3
......................uggaucuuguucgacagcU	164	1	LT-3
......................uggaucuuguucgCcagca	2	1	LT-1
......................uggaucuuguucgacagca	294	0	JT-3
......................uggCucuuguucgacagca	1	1	JCK-1
......................uggaucuuguucgacagca	1184	0	LT-2
......................uggauUuuguucgacagca	1	1	JCK-3
......................uggaucuuguucgacagca	932	0	JCK-2
......................uggaucuuguucgacagUa	9	1	LCK-3
......................uggaucuuguucgacGgca	1	1	JT-1
......................uggaucuuguucgacagca	237	0	JT-2
......................uggaucuuguucNacagca	1	1	LCK-1
......................uggaucuuguucgacagUa	6	1	JCK-2
......................uggaucuuguucgacagUa	5	1	LT-1
......................uggaucuuguucgCcagca	5	1	LCK-1
......................ugAaucuuguucgacagcac	2	1	JCK-1
......................uggaucuuguucgacagcUc	3	1	LT-2
......................uggaucuuguucgacagcaA	1	1	JCK-1
......................Cggaucuuguucgacagcac	2	1	LT-2
......................ugUaucuuguucgacagcac	1	1	JCK-3
......................uggaucuuguucgCcagcac	1	1	LT-3
......................uggCucuuguucgacagcac	11	1	LT-2
......................uggaucuuguucgacagcac	762	0	LT-3
......................uggaucuuguucgacagcUc	2	1	JCK-3
......................uggCucuuguucgacagcac	2	1	LT-3
......................uggaucuuguucgaAagcac	1	1	LT-3
......................uggaucuuguucgacagcac	83	0	JT-3
......................uggaucuuguucgNcagcac	4	1	LCK-1
......................uggaucuuguucgacagcac	508	0	JCK-3
......................uggaucuuguucgacagcCc	1	1	LCK-2
......................uggaucuuguucgacCgcac	2	1	JCK-1
......................ugAaucuuguucgacagcac	1	1	LT-2
......................uggaucuuguucgCcagcac	1	1	LCK-2
......................uggaucuuguucgacagcaA	3	1	JCK-2
......................uggCucuuguucgacagcac	1	1	JT-3
......................uggCucuuguucgacagcac	2	1	LCK-1
......................uggaucuuguucgCcagcac	5	1	LT-2
......................uggaucuuguucgacCgcac	1	1	LT-2
......................uggaucuuguucgacagcaU	5	1	JCK-1
......................uggaucuuguucgacGgcac	1	1	LT-1
......................uggaCcuuguucgacagcac	1	1	LCK-3
......................uggaucuuguucAacagcac	1	1	LT-2
......................uggaucuuguucgacagcac	164	0	JT-1
......................uggaucuuguucgaUagcac	3	1	JCK-2
......................uggaucuuguucgacagcac	1028	0	LT-2
......................ugUaucuuguucgacagcac	1	1	LT-3
......................uggaucuuguucgacagcaU	12	1	JCK-3
......................uggaucuuguucgaUagcac	1	1	JCK-1
......................uggCucuuguucgacagcac	5	1	LCK-3
......................uggaucuuguucgGcagcac	1	1	JCK-1
......................uggaucuuguucgacagcaU	16	1	LT-3
......................uggaucuuguucgacagcaA	2	1	LT-3
......................uggaucuuguucgacagcaA	9	1	JCK-3
......................uggaucuuguucgCcagcac	6	1	LCK-3
......................uAgaucuuguucgacagcac	1	1	LT-3
......................uggaucuuguucgGcagcac	1	1	LCK-1
......................uggaucuuguucgaAagcac	1	1	JCK-1
......................ugAaucuuguucgacagcac	1	1	LCK-3
......................uggCucuuguucgacagcac	2	1	JCK-2
......................uggaucuuguucgacagcac	2109	0	LCK-3
......................uggaucuuguucgacagcCc	3	1	LT-2
......................uggaucuuguucgacagUac	1	1	JCK-3
......................uggaucuuguucgacagcac	72	0	JT-2
......................uggaucuuguucgacagcaU	11	1	LT-2

```
                      Mature                                                     Star
uccaucuuguucguuuacccucuggaucuuguucgacagcacucucuccuaucuuguuugacuacugaugaugagagugcugucgacaaaauccagaaggugcugucgaauaagauacagauacagaggaacgagcaacuguaccuacugucaugacaaucuucuucauauucucuucaauuuuuauuuauuuuuuuucuuauuguucguguuuuauuuuuuuuuuuaauuuccagauuagauuuaccu
......................uggaucuuguuNgacagcac.      1     1     JT-2
......................uggaucuuguucgacagcUc.      1     1     LT-1
......................uggaucuuguucgacagcCc.      1     1     LT-1
......................ugNaucuuguucgacagcac.      4     1     LT-2
......................uggaucuuguucgacagcUc.      2     1     JCK-2
......................uggaucuuguucgacaAcac.      1     1     JCK-1
......................uggaucuuguucgaUagcac.      1     1     LT-2
......................uggaucuuguucgacagcGc.      1     1     LT-2
......................uggaucuuAuucgacagcac.      1     1     LCK-1
......................uggaucuuguucgacagcac.      564   0     LT-1
......................uggaucuuguucgacagcac.      796   0     LCK-2
......................uggaucuuguucgacagcac.      821   0     LCK-1
......................uggaucuuguucgaNagcac.      4     1     LCK-1
......................uggauNuuguucgacagcac.      2     1     LCK-1
......................uggaucuuguucgaUagcac.      3     1     JCK-3
......................uggaucuuguucgacagcaU.      1     1     LCK-3
......................uggaucuuguucgacagcaU.      10    1     LT-1
......................uggaucuuguucgacagcCc.      1     1     JT-3
......................uAgaucuuguucgacagcac.      1     1     LCK-3
......................uggaucuuguucgacGgcac.      1     1     LT-2
......................uggaucuuguucgacagcUc.      3     1     LT-3
......................uggaucuuguucgacagcac.      454   0     JCK-1
......................uggaucuugCucgacagcac.      1     1     LT-2
......................uggaucuuguucgacagcNc.      1     1     LCK-1
......................uggaucuuguucgacagcNc.      1     1     JCK-1
......................uggaucuuguucUacagcac.      1     1     LT-1
......................uggCucuuguucgacagcac.      4     1     LCK-2
......................uNgaucuuguucgacagcac.      1     1     JCK-1
......................uggaucuuguucgacGgcac.      1     1     JCK-2
......................uggaucuuguucgaGagcac.      1     1     LCK-1
......................uggaucuuguucgaAagcac.      2     1     LCK-2
......................ugUaucuuguucgacagcac.      1     1     LT-2
......................uggaucuuguucgacagcaU.      19    1     JCK-2
......................uggCucuuguucgacagcac.      1     1     LT-1
......................uggaucuuguucgCcagcac.      1     1     JCK-1
......................uggaucuuguucgacagcUc.      1     1     JCK-1
......................uggaucuuguucgacagcac.      727   0     JCK-2
......................uggaucuuguucgacagcCc.      1     1     LCK-3
......................uggaucuuguuAgacagcacu.     1     1     JCK-1
......................uggaCcuuguucgacagcacu.     1     1     JCK-1
......................uggaucuuguuGgacagcacu.     10    1     JCK-1
......................uggaucuuguucgacagcacA.     61    1     LCK-3
......................uggaucuuguuGgacagcacu.     1     1     JCK-2
......................uggaucuuguucgacaAcacu.     8     1     JCK-1
......................ugNaucuuguucgacagcacu.     9     1     LT-2
......................uggaucCuguucgacagcacu.     1     1     JT-1
......................uggaucuuguucgacagcacG.     2     1     LT-3
......................uggaucuuguucgacaUcacu.     5     1     JCK-2
......................uggaucuuguucgacagcacA.     51    1     JCK-1
......................uggaucuuguucgNcagcacu.     5     1     LCK-1
......................uggaucuuguucgacGgcacu.     2     1     JCK-1
......................Aggaucuuguucgacagcacu.     1     1     JCK-2
......................uggNucuuguucgacagcacu.     21    1     JCK-1
......................uggaucuuguucgaUagcacu.     12    1     JCK-1
......................uggaucuuguucgacagcacC.     8     1     LT-2
......................uggaucuuguucgacagcaAu.     3     1     JCK-2
......................Gggaucuuguucgacagcacu.     1     1     JCK-3
......................Cggaucuuguucgacagcacu.     8     1     JCK-2
......................uggaucuuguucgacagcacu.     4196  0     LT-2
......................uggaucuuguucgacagcacA.     79    1     JCK-2
......................uggaucGuguucgacagcacu.     1     1     JCK-3
......................uggaucuuAuucgacagcacu.     10    1     JCK-2
......................uggaucuuguucgacGgcacu.     1     1     LCK-3
......................uggaucuuguucgacagcCcu.     1     1     LCK-1
......................uggaucuuguucgacagUacu.     1     1     LT-1
......................uUgaucuuguucgacagcacu.     3     1     JCK-2
......................ugAaucuuguucgacagcacu.     2     1     LCK-3
......................uggGucuuguucgacagcacu.     9     1     JCK-1
......................uggaCcuuguucgacagcacu.     5     1     JCK-2
......................uggCucuuguucgacagcacu.     75    1     JCK-3
```

Mature **Star**

uccaucuuguucguuuacccucuggaucuuguucgacagcacucucuccuaucuuguuugacuacugaugaugagagugcugucgacaaaauccagaaggugcugucgaauaagauacagauacagaggaacgagcaacuguaccuacugucaugacaaucuucuucauauucucuucaauuuuuauuuauuuuuuuucuuauuguucguguuuuauuuuuuuuuuuaauuuccagauuagauuuaccu

uNgaucuuguucgacagcacu	29	1	JCK-3
uggaucuuguucgacagcGcu	2	1	JCK-3
uggaucuuguucgacagcCcu	3	1	LT-1
uggGucuuguucgacagcacu	1	1	LCK-3
uggauUuuguucgacagcacu	4	1	JCK-3
Aggaucuuguucgacagcacu	4	1	LT-2
uggaucuuguuAgacagcacu	1	1	JCK-2
uggaucuuguucgacGgcacu	1	1	LT-2
uggaucuuguucgacagcacu	5544	0	LCK-3
uggaucuuguucgacaAcacu	2	1	JCK-3
uggaucuuguucgCcagcacu	16	1	LT-2
uggNucuuguucgacagcacu	4	1	JCK-3
uggaucuuguucgGcagcacu	2	1	LCK-1
uggaucuuguGcgacagcacu	3	1	JCK-1
uggaucuuguucgaUagcacu	2	1	LT-3
uggaucuuguucgacCgcacu	7	1	JCK-1
uggaucuuguucUacagcacu	6	1	JCK-3
uggaucuuguucgaAagcacu	5	1	JCK-2
uggaucCuguucgacagcacu	5	1	JCK-3
uggaucuuguucgacGgcacu	5	1	JCK-2
uggaucuuguucgacagcacC	1	1	LT-1
uggaucuuguucgacagcaUu	18	1	LT-1
uggauNuuguucgacagcacu	1	1	JCK-1
ugAaucuuguucgacagcacu	8	1	JCK-2
uggaucuuAuucgacagcacu	1	1	LT-3
uggaucuuguuNgacagcacu	1	1	JCK-2
uggaucuuguucgacagcacG	2	1	LT-1
uggaucuuguuNgacagcacu	1	1	JT-1
uggaucuuguucgacagcacC	4	1	JT-3
uggaucuuguucgacagUacu	16	1	JCK-1
uggaucuuguucgacagcUcu	1	1	JCK-3
uggaucuuAuucgacagcacu	1	1	LT-2
uggaucuuguucgacaAcacu	1	1	LT-1
uggaucuuguucgacagcacu	4453	0	LT-3
uAgaucuuguucgacagcacu	1	1	JT-1
uggaucCuguucgacagcacu	2	1	LT-2
uggCucuuguucgacagcacu	19	1	LT-3
uggaucuuguucgacagcacA	16	1	LT-1
uggaucuuguucgacagcacu	423	0	JT-3
ugNaucuuguucgacagcacu	2	1	LCK-1
uggaucuuguucgacagcacA	24	1	LT-3
uggaucuuguucgCcagcacu	6	1	LCK-3
uggaucuuguucgacaAcacu	1	1	LCK-3
uggaucuuguucNacagcacu	1	1	LCK-1
uggaucuuguucgaUagcacu	2	1	LT-1
uggaucuuguucgacagcCcu	17	1	JCK-3
uggaucuuguucgacagcaGu	1	1	JCK-1
uggaucCuguucgacagcacu	7	1	JCK-2
uggaucuuguucAacagcacu	1	1	LCK-1
ugAaucuuguucgacagcacu	1	1	LT-3
uggaucuuguucgacagcCcu	1	1	JT-1
uggCucuuguucgacagcacu	1	1	JT-2
uggaucuuguucgacagcaUu	38	1	JCK-2
uggaucuuguucgaUagcacu	11	1	JCK-3
uggaucuuguucgacagcaAu	3	1	LT-1
uggaucuuguucgacagcGcu	1	1	LT-3
uUgaucuuguucgacagcacu	2	1	LCK-3
uggaucuCguucgacagcacu	1	1	LT-1
uggaucuuguucgacagcacC	10	1	JCK-3
uggaucuuguucgacagcacA	59	1	JCK-3
uggaucuuguucgacagcaUu	7	1	JT-1
ugAaucuuguucgacagcacu	2	1	LT-2
uggauUuuguucgacagcacu	4	1	JCK-2
uggaCcuuguucgacagcacu	1	1	LCK-2
uggaucuCguucgacagcacu	1	1	JCK-1
uggaucuuNuucgacagcacu	2	1	JCK-3
uggaucuuguucgacagcacA	37	1	JT-1
uAgaucuuguucgacagcacu	7	1	JCK-3
uggaucuuguucgacCgcacu	10	1	JCK-3
uggNucuuguucgacagcacu	1	1	JCK-2

```
                      Mature                                                    Star
uccaucuuguucguuuacccucuggaucuuguucgacagcacucucuccuaucuuguuugacuacugaugaugagagugcugucgacaaaauccagaaggugcugucgaauaagauacagauacagaggaacgagcaacuguaccuacugucaugacaaucuucuucauauucucuucaauuuuuauuuauuuuuuuucuuauuguucguguuuuauuuuuuuuuuuaauuuccagauuagauuuaccu
......................uggaucuuguucgacagcaUu..............................................................................................................................................................................    17      1    JCK-3
......................uAgaucuuguucgacagcacu..............................................................................................................................................................................    1       1    LT-1
......................uggCucuuguucgacagcacu..............................................................................................................................................................................    55      1    LT-2
......................uggCucuuguucgacagcacu..............................................................................................................................................................................    10      1    LT-1
......................uggaucuuguucgacagcGcu..............................................................................................................................................................................    7       1    JCK-2
......................uUgaucuuguucgacagcacu..............................................................................................................................................................................    6       1    JCK-3
......................ugAaucuuguucgacagcacu..............................................................................................................................................................................    2       1    LT-1
......................ugAaucuuguucgacagcacu..............................................................................................................................................................................    6       1    JCK-1
......................ugNaucuuguucgacagcacu..............................................................................................................................................................................    1       1    LT-1
......................uggaucuugCucgacagcacu..............................................................................................................................................................................    3       1    JCK-3
......................uggaucuuguucgacagcacu..............................................................................................................................................................................    1314    0    LCK-2
......................uAgaucuuguucgacagcacu..............................................................................................................................................................................    3       1    LT-2
......................uggaucuuguucgacagUacu..............................................................................................................................................................................    19      1    JCK-3
......................uggaucuuguucgacGgcacu..............................................................................................................................................................................    1       1    LT-1
......................uggaucCuguucgacagcacu..............................................................................................................................................................................    2       1    JCK-1
......................ugUaucuuguucgacagcacu..............................................................................................................................................................................    3       1    JCK-2
......................uggaucuuguucgacagcaAu..............................................................................................................................................................................    1       1    LCK-2
......................uCgaucuuguucgacagcacu..............................................................................................................................................................................    3       1    JCK-2
......................uggaucuuguucgaAagcacu..............................................................................................................................................................................    1       1    JCK-1
......................uggaucuuguucgacCgcacu..............................................................................................................................................................................    13      1    JCK-2
......................uggaucuuguucAacagcacu..............................................................................................................................................................................    1       1    JCK-3
......................uggaucuuguucCacagcacu..............................................................................................................................................................................    4       1    LCK-3
......................uggaucuuguucgacGgcacu..............................................................................................................................................................................    1       1    LCK-2
......................uggaucuuguucgacagcaNu..............................................................................................................................................................................    23      1    JCK-1
......................ugUaucuuguucgacagcacu..............................................................................................................................................................................    7       1    JCK-3
......................uggaucuuguucgacagcaAu..............................................................................................................................................................................    12      1    JCK-1
......................Cggaucuuguucgacagcacu..............................................................................................................................................................................    2       1    LT-3
......................uggaucuugCucgacagcacu..............................................................................................................................................................................    9       1    JCK-2
......................uggaucuuguucgacagcacG..............................................................................................................................................................................    15      1    JCK-2
......................uggaucuuguucgacagcacC..............................................................................................................................................................................    3       1    LT-3
......................uggaucuGguucgacagcacu..............................................................................................................................................................................    6       1    JCK-2
......................uggaucuuAuucgacagcacu..............................................................................................................................................................................    2       1    JCK-1
......................uggaucuuguucgacagcacC..............................................................................................................................................................................    2       1    JT-2
......................uggGucuuguucgacagcacu..............................................................................................................................................................................    4       1    JCK-3
......................ugUaucuuguucgacagcacu..............................................................................................................................................................................    1       1    LT-1
......................uggaucuuAuucgacagcacu..............................................................................................................................................................................    1       1    JCK-3
......................ugUaucuuguucgacagcacu..............................................................................................................................................................................    2       1    JCK-1
......................uggaucuuguucgacagcCcu..............................................................................................................................................................................    2       1    LCK-3
......................uggaucuuguucgacagcacA..............................................................................................................................................................................    50      1    LT-2
......................uggaucuuguucgacagcCcu..............................................................................................................................................................................    7       1    LT-2
......................uggaucuuguucgacaCcacu..............................................................................................................................................................................    1       1    JCK-1
......................uggaucuuguucgacagNacu..............................................................................................................................................................................    3       1    JCK-3
......................uggaucuuguucgacagcacu..............................................................................................................................................................................    20810   0    JCK-1
......................uggaucuCguucgacagcacu..............................................................................................................................................................................    7       1    JCK-2
......................uggaucuuguucgGcagcacu..............................................................................................................................................................................    2       1    JCK-3
......................uggaucuuguucAacagcacu..............................................................................................................................................................................    7       1    JCK-2
......................uggaucuuguucgacagAacu..............................................................................................................................................................................    1       1    JCK-2
......................uggaucuuguucgacagcNcu..............................................................................................................................................................................    10      1    JCK-1
......................uggaucuuguCcgacagcacu..............................................................................................................................................................................    1       1    JCK-3
......................uAgaucuuguucgacagcacu..............................................................................................................................................................................    10      1    JCK-2
......................uggaucuuguucgacagcacC..............................................................................................................................................................................    22      1    JCK-2
......................Cggaucuuguucgacagcacu..............................................................................................................................................................................    1       1    LCK-3
......................uggaucuuguucgacagcCcu..............................................................................................................................................................................    11      1    JCK-1
......................uggaucuuguucgacagUacu..............................................................................................................................................................................    23      1    JCK-2
......................uggaucuugCucgacagcacu..............................................................................................................................................................................    6       1    LCK-3
......................uggaucuuguucgacagcaAu..............................................................................................................................................................................    2       1    JCK-3
......................uggaucuuguucgCcagcacu..............................................................................................................................................................................    47      1    JCK-1
......................uggaucuuguucgCcagcacu..............................................................................................................................................................................    8       1    LT-1
......................uggaucuugCucgacagcacu..............................................................................................................................................................................    3       1    JCK-1
......................uggaucuuguuNgacagcacu..............................................................................................................................................................................    121     1    JCK-1
......................uggaucuuguucgacagGacu..............................................................................................................................................................................    7       1    JCK-1
......................uggaCcuuguucgacagcacu..............................................................................................................................................................................    1       1    LCK-3
......................uggaucuuguucgaUagcacu..............................................................................................................................................................................    26      1    JCK-2
......................uggaucuuguucgacagcacu..............................................................................................................................................................................    26265   0    JCK-3
......................uggaucuuguuAgacagcacu..............................................................................................................................................................................    1       1    LCK-3
......................ugAaucuuguucgacagcacu..............................................................................................................................................................................    11      1    JCK-3
......................uggaucAuguucgacagcacu..............................................................................................................................................................................    1       1    JCK-2
......................uggaucuuUuucgacagcacu..............................................................................................................................................................................    3       1    JCK-2
......................uggaucuuCuucgacagcacu..............................................................................................................................................................................    1       1    JCK-3
......................uggaucuuguucgacGgcacu..............................................................................................................................................................................    10      1    JCK-3
```

Mature Star

```
uccaucuuguucguuuacccucuggaucuuguucgacagcacucucuccuaucuuguuugacuacugaugaugagagugcugucgacaaaauccagaaggugcugucgaauaagauacagauacagaggaacgagcaacuguaccuacugucaugacaaucuucuucauauucucuucaauuuuuauuuauuuuuuuucuuauuguucguguuuuauuuuuuuuuuuaauuuccagauuagauuuaccu
......................uggaucuuguucgacagcaAu.......... 1 1 LCK-3
......................uNgaucuuguucgacagcacu.......... 31 1 JCK-1
......................uggNucuuguucgacagcacu.......... 1 1 LCK-1
......................ugCaucuuguucgacagcacu.......... 1 1 JCK-1
......................uggaucuuguucgacagcaUu.......... 17 1 LT-3
......................uggaucuuguucgCcagcacu.......... 2 1 LCK-2
......................uggaucuuguucgacagcaAu.......... 2 1 LT-3
......................uggaucuuguucAacagcacu.......... 1 1 LCK-2
......................uggaucuuguucgCcagcacu.......... 80 1 JCK-2
......................uAgaucuuguucgacagcacu.......... 2 1 LCK-2
......................uggaucuuguucgacCgcacu.......... 3 1 LT-3
......................uggaCcuuguucgacagcacu.......... 4 1 JCK-3
......................uggCucuuguucgacagcacu.......... 2 1 LCK-2
......................uggaucuCguucgacagcacu.......... 1 1 LCK-2
......................uggaucuuguucgCcagcacu.......... 48 1 JCK-3
......................uggaucuuguCcgacagcacu.......... 2 1 JCK-1
......................uAgaucuuguucgacagcacu.......... 9 1 JCK-1
......................uggaucuuguucgacagcacG.......... 12 1 JCK-3
......................uggaucuuguucgacagcacN.......... 6 1 JCK-1
......................uggCucuuguucgacagcacu.......... 1 1 LCK-1
......................uggaucuuguucgacagcaAu.......... 3 1 LT-2
......................uggaucuCguucgacagcacu.......... 2 1 JCK-3
......................uggaucuuguuGgacagcacu.......... 1 1 JCK-3
......................uggaucuuguucgacaUcacu.......... 1 1 LT-2
......................uggaucuuUuucgacagcacu.......... 3 1 JCK-3
......................uggaucuuguuGgacagcacu.......... 1 1 LT-3
......................uNgaucuuguucgacagcacu.......... 2 1 LT-3
......................uAgaucuuguucgacagcacu.......... 3 1 LT-3
......................uggCucuuguucgacagcacu.......... 67 1 JCK-1
......................uggaucuuguucgacagcCcu.......... 24 1 JCK-2
......................uggaucuuguCcgacagcacu.......... 1 1 LT-3
......................uggaucuuguucgGcagcacu.......... 2 1 JCK-1
......................uggaucuuguucgCcagcacu.......... 10 1 LT-3
......................uggaucuuguucgacagcUcu.......... 1 1 JCK-2
......................uggaucuuguucgaGagcacu.......... 1 1 JCK-3
......................uAgaucuuguucgacagcacu.......... 7 1 LCK-3
......................uggGucuuguucgacagcacu.......... 3 1 JCK-2
......................uUgaucuuguucgacagcacu.......... 1 1 LT-2
......................uggaucuuguucgacagcacu.......... 277 0 JT-2
......................uggaucuuguucgCcagcacu.......... 2 1 JT-1
......................uCgaucuuguucgacagcacu.......... 1 1 LT-2
......................uggaucuuguucgacCgcacu.......... 3 1 LT-2
......................uggaucuuguucgCcagcacu.......... 2 1 JT-3
......................uggaucCuguucgacagcacu.......... 1 1 JT-3
......................uggaucuuguucgacagcacG.......... 6 1 LCK-3
......................uggaucuuguucgacagcacC.......... 5 1 JCK-1
......................uggaucuuguucgaAagcacu.......... 9 1 JCK-3
......................uggaucuuguucgacagcGcu.......... 1 1 JCK-1
......................uggaucuuguucgacagcacu.......... 1125 0 JT-1
......................Cggaucuuguucgacagcacu.......... 2 1 LT-2
......................uggaucuuguucgacagcacu.......... 2933 0 LT-1
......................uCgaucuuguucgacagcacu.......... 1 1 JCK-3
......................uggauUuuguucgacagcacu.......... 1 1 JCK-1
......................ugUaucuuguucgacagcacu.......... 1 1 LCK-3
......................uggaucuuguucCacagcacu.......... 1 1 JCK-2
......................uggaucuuguucgacaUcacu.......... 2 1 JCK-1
......................uggaucuuguucgacagcacG.......... 12 1 JCK-1
......................uggaucuuguucgacCgcacu.......... 1 1 LCK-2
......................uggaucuuguucgacagcUcu.......... 2 1 LT-1
......................uggaucuuguucAacagcacu.......... 6 1 JCK-1
......................uggaucuuguCcgacagcacu.......... 2 1 JCK-2
......................uUgaucuuguucgacagcacu.......... 1 1 JCK-1
......................uggaucuuguucgacagcacu.......... 1264 0 LCK-1
......................uggaucuuguucgacagcCcu.......... 2 1 LT-3
......................uggaucCuguucgacagcacu.......... 2 1 LCK-3
......................uggauNuuguucgacagcacu.......... 1 1 LCK-1
......................uggCucuuguucgacagcacu.......... 4 1 JT-1
......................uggaucuuguucgacaAcacu.......... 9 1 JCK-2
......................uggaucuuguucgacagcaUu.......... 13 1 JCK-1
......................uggaucuuguucgacagcCcu.......... 1 1 LCK-2
```

```
                      Mature                                                      Star
uccaucuuguucguuuacccucuggaucuuguucgacagcacucucuccuaucuuguuugacuacugaugaugagagugcugucgacaaaauccagaaggugcugucgaauaagauacagauacagaggaacgagcaacuguaccuacugucaugacaaucuucuucauauucucuucaauuuuuauuuauuuuuuuucuuauuguucguguuuuauuuuuuuuuuuuaauuuccagauuagauuuaccu
......................uggaucuuguucgacagcacu...     35956   0   JCK-2
......................Cggaucuuguucgacagcacu...     2       1   JCK-3
......................uggaucuuguucUacagcacu...     4       1   JCK-2
......................uggaucuuguucgacagcacC...     2       1   LCK-3
......................uggaucuuguucgacaUcacu...     4       1   JCK-3
......................uggaucuuguucgaNagcacu...     4       1   LCK-1
......................uggaucuuAuucgacagcacu...     3       1   LCK-3
......................uggaucuuguucgacagcaUu...     38      1   LCK-3
......................Cggaucuuguucgacagcacu...     2       1   JCK-1
......................uggCucuuguucgacagcacu...     104     1   JCK-2
......................uggaucuuguucgacagcaUu...     17      1   LT-2
......................uggCucuuguucgacagcacu...     18      1   LCK-3
......................uggaucuuguucUacagcacu...     1       1   JCK-1
......................uNgaucuuguucgacagcacu...     37      1   JCK-2
......................uggaucuuguucgacaCcacuc...    1       1   LT-1
......................uggaucuuguucgacagGacuc...    2       1   JCK-2
......................uggaucuuUuucgacagcacuc...    3       1   JCK-2
......................uggaucuuguucgUcagcacuc...    1       1   JCK-1
......................uggaucuuAuucgacagcacuc...    1       1   LT-3
......................uNgaucuuguucgacagcacuc...    1       1   LT-3
......................uUgaucuuguucgacagcacuc...    1       1   LT-1
......................uggaucuuguucgacaAcacuc...    1       1   LT-1
......................uggCucuuguucgacagcacuc...    19      1   JCK-1
......................uNgaucuuguucgacagcacuc...    15      1   JCK-2
......................uggaCcuuguucgacagcacuc...    3       1   JCK-3
......................uggaucuuguucgCcagcacuc...    11      1   JCK-3
......................uggaucuuguucgacagcacuG...    2       1   JCK-3
......................uggaucuuguucgacCgcacuc...    8       1   JCK-2
......................ugAaucuuguucgacagcacuc...    5       1   LT-3
......................uggaucuuUuucgacagcacuc...    3       1   JCK-3
......................uggCucuuguucgacagcacuc...    7       1   LT-3
......................uggaucuuguucCacagcacuc...    2       1   JCK-1
......................uggaucuuAuucgacagcacuc...    1       1   JCK-3
......................uggaucuuguucUacagcacuc...    1       1   JCK-2
......................uggaucuuguucgacagcacuU...    25      1   LT-2
......................uggaucuuguucgacagcacuc...    10812   0   JCK-3
......................uggaucuuguuGgacagcacuc...    3       1   JCK-1
......................uggaCcuuguucgacagcacuc...    2       1   JCK-2
......................uggaucuuguucgacagcacuU...    1       1   LCK-1
......................Cggaucuuguucgacagcacuc...    2       1   JCK-1
......................uggaucuuguucgacagcaUuc...    1       1   LT-1
......................uggaucuCguucgacagcacuc...    1       1   LCK-3
......................uggaucuuguucgacGgcacuc...    1       1   JCK-1
......................uggaucuuguucgaUagcacuc...    1       1   LT-3
......................uggaucuuguucgacagcacuc...    2355    0   LT-3
......................uggaucuuguucgCcagcacuc...    1       1   LT-2
......................uggaucuuguucgacCgcacuc...    5       1   JCK-3
......................uggaucuuguucgacagcacuc...    69      0   JT-3
......................uggaucuuguucgacagcacuA...    14      1   LCK-1
......................uggaucuuguucgacaUcacuc...    1       1   JCK-3
......................ugAaucuuguucgacagcacuc...    5       1   JCK-3
......................uggaucuuguucgacagcacGc...    1       1   JCK-1
......................uggaucuuguucgacagcaUuc...    10      1   JCK-3
......................uggaucuuguucgacagcacuU...    27      1   LT-1
......................uggaucuuguucUacagcacuc...    2       1   LT-1
......................uggaucuuguucgacagcacuG...    9       1   JCK-1
......................uggaucuuguucgacagcacuc...    84      0   LCK-2
......................uggaucuuguucgacagcacuG...    3       1   LT-1
......................uggaucuuguucgacaUcacuc...    4       1   JCK-2
......................ugAaucuuguucgacagcacuc...    2       1   JCK-1
......................uggaucuuguucgacGgcacuc...    1       1   LT-1
......................uggaucuuguucgacagNacuc...    1       1   JCK-3
......................uggaucuugCucgacagcacuc...    2       1   LT-1
......................uggaucuuguucgacagcacCc...    1       1   LT-3
......................uggGucuuguucgacagcacuc...    2       1   JCK-2
......................uggaucuuguucgaUagcacuc...    42      1   JCK-2
......................uggCucuuguucgacagcacuc...    43      1   JCK-2
......................uggaucuuguucAacagcacuc...    1       1   LT-1
......................uggaucuuguucgacagUacuc...    3       1   JCK-3
......................uggaucuuguucgacCgcacuc...    1       1   LT-3
```

```
                      Mature                           Star
uccaucuuguucguuuacccucuggaucuuguucgacagcacucucuccuaucuuguuugacuacugaugaugagagugcugucgacaaaauccagaaggugcugucgaauaagauacagauacagaggaacgagcaacuguaccuacugucaugacaaucuucuucauauucucuucaauuuuuauuuauuuuuuuucuuauuguucguguuuuauuuuuuuuuuuaauuuccagauuagauuuaccu
......................uggaucuuguGcgacagcacuc.............................................................................................................................................................................................................	5	1	JCK-1
......................uggCucuuguucgacagcacuc.............................................................................................................................................................................................................	24	1	JCK-3
......................uggaucuuguucgacagcacuA.............................................................................................................................................................................................................	36	1	LCK-3
......................Cggaucuuguucgacagcacuc.............................................................................................................................................................................................................	1	1	LT-2
......................uggaucuuguucgaAagcacuc.............................................................................................................................................................................................................	5	1	JCK-2
......................uggaucuuguucAacagcacuc.............................................................................................................................................................................................................	2	1	JCK-3
......................uggaucuuguucAacagcacuc.............................................................................................................................................................................................................	1	1	JCK-2
......................uggaucuuguucgacagcaUuc.............................................................................................................................................................................................................	7	1	JCK-1
......................uggCucuuguucgacagcacuc.............................................................................................................................................................................................................	3	1	LT-1
......................uggaucuuguucgacGgcacuc.............................................................................................................................................................................................................	4	1	JCK-3
......................uggaucuuUuucgacagcacuc.............................................................................................................................................................................................................	1	1	JCK-1
......................uggaucuuNuucgacagcacuc.............................................................................................................................................................................................................	1	1	JCK-3
......................uAgaucuuguucgacagcacuc.............................................................................................................................................................................................................	3	1	JCK-1
......................uggaucuuguucgacagcNcuc.............................................................................................................................................................................................................	3	1	JCK-1
......................uggCucuuguucgacagcacuc.............................................................................................................................................................................................................	1	1	LCK-3
......................uggaucuuguucgacagcacuU.............................................................................................................................................................................................................	63	1	JCK-1
......................uggaucuuguucUacagcacuc.............................................................................................................................................................................................................	2	1	JCK-3
......................uggaucuuguucgacagcCcuc.............................................................................................................................................................................................................	1	1	LT-2
......................ugNaucuuguucgacagcacuc.............................................................................................................................................................................................................	2	1	LT-2
......................uggaucuuguucgacagcacuN.............................................................................................................................................................................................................	4	1	JCK-1
......................uggaucuuguucgacCgcacuc.............................................................................................................................................................................................................	1	1	LCK-2
......................uggNucuuguucgacagcacuc.............................................................................................................................................................................................................	8	1	JCK-1
......................uggaucuuguucgacagcacuU.............................................................................................................................................................................................................	31	1	LCK-3
......................uggaucuuguucgacagcacuc.............................................................................................................................................................................................................	1033	0	LT-2
......................uggaucuuguucgacagcacAc.............................................................................................................................................................................................................	1	1	LT-1
......................uggaucuuAuucgacagcacuc.............................................................................................................................................................................................................	2	1	JCK-2
......................Cggaucuuguucgacagcacuc.............................................................................................................................................................................................................	3	1	JCK-2
......................uggaucuuguucgUcagcacuc.............................................................................................................................................................................................................	3	1	JCK-2
......................uggaucuuguucgacagcacuG.............................................................................................................................................................................................................	12	1	JCK-2
......................uggaucCuguucgacagcacuc.............................................................................................................................................................................................................	3	1	JCK-3
......................uggaucuCguucgacagcacuc.............................................................................................................................................................................................................	1	1	JCK-3
......................uggaucuuguucgacagcaNuc.............................................................................................................................................................................................................	5	1	JCK-1
......................uggaCcuuguucgacagcacuc.............................................................................................................................................................................................................	1	1	JCK-1
......................uggaucuuguucgCcagcacuc.............................................................................................................................................................................................................	29	1	JCK-2
......................uggaucuuguucgCcagcacuc.............................................................................................................................................................................................................	17	1	JCK-1
......................uggaucuuguucgacCgcacuc.............................................................................................................................................................................................................	3	1	JCK-1
......................uAgaucuuguucgacagcacuc.............................................................................................................................................................................................................	3	1	JCK-3
......................uggaucuuguucgGcagcacuc.............................................................................................................................................................................................................	1	1	LCK-1
......................uggaucuuAuucgacagcacuc.............................................................................................................................................................................................................	1	1	JCK-1
......................uggaucuCguucgacagcacuc.............................................................................................................................................................................................................	1	1	LT-1
......................uggaucuuguucgCcagcacuc.............................................................................................................................................................................................................	5	1	LT-3
......................uggaucuuguucgacagUacuc.............................................................................................................................................................................................................	4	1	JCK-1
......................uggaucuuguucgCcagcacuc.............................................................................................................................................................................................................	2	1	LT-1
......................uggauUuuguucgacagcacuc.............................................................................................................................................................................................................	4	1	JCK-2
......................uggaucuuguucAacagcacuc.............................................................................................................................................................................................................	1	1	JCK-1
......................uggaucuuguucgacagcCcuc.............................................................................................................................................................................................................	9	1	JCK-2
......................uggaucuuguucgacagcacuc.............................................................................................................................................................................................................	58	0	JT-2
......................uggauUuuguucgacagcacuc.............................................................................................................................................................................................................	3	1	JCK-1
......................uggGucuuguucgacagcacuc.............................................................................................................................................................................................................	4	1	JCK-1
......................uggaucuuguucgacaUcacuc.............................................................................................................................................................................................................	1	1	JCK-1
......................uggaucuuguucgUcagcacuc.............................................................................................................................................................................................................	1	1	JCK-3
......................uggaucuuguucAacagcacuc.............................................................................................................................................................................................................	1	1	LT-2
......................uggaucuuguuNgacagcacuc.............................................................................................................................................................................................................	29	1	JCK-1
......................uggaucCuguucgacagcacuc.............................................................................................................................................................................................................	1	1	JCK-2
......................uggaucuuguucgacagcCcuc.............................................................................................................................................................................................................	2	1	LT-1
......................uggaucCuguucgacagcacuc.............................................................................................................................................................................................................	2	1	LCK-3
......................uggaucuuguucgaUagcacuc.............................................................................................................................................................................................................	10	1	JCK-1
......................uggaCcuuguucgacagcacuc.............................................................................................................................................................................................................	1	1	LT-1
......................ugAaucuuguucgacagcacuc.............................................................................................................................................................................................................	4	1	JCK-2
......................uggaucuuguCcgacagcacuc.............................................................................................................................................................................................................	1	1	JCK-3
......................uggaucuugCucgacagcacuc.............................................................................................................................................................................................................	4	1	JCK-3
......................uggaucuuguucgacagGacuc.............................................................................................................................................................................................................	6	1	JCK-1
......................uggaucuuguucgacagcacNc.............................................................................................................................................................................................................	4	1	JCK-1
......................uggaucuuguucgacagcCcuc.............................................................................................................................................................................................................	1	1	LT-3
......................uggaucuuguucgacagcacAc.............................................................................................................................................................................................................	1	1	JCK-2
......................uggauUuuguucgacagcacuc.............................................................................................................................................................................................................	2	1	JCK-3
......................uggaucuuguucgacagcacuA.............................................................................................................................................................................................................	61	1	JCK-2
......................uAgaucuuguucgacagcacuc.............................................................................................................................................................................................................	2	1	LT-1
......................uggaucuugCucgacagcacuc.............................................................................................................................................................................................................	5	1	JCK-1
......................uggaucCuguucgacagcacuc.............................................................................................................................................................................................................	1	1	LT-3
```

```
                      Mature                                                Star
uccaucuuguucguuuacccucuggaucuuguucgacagcacucucuccuaucuuguuugacuacugaugaugagagugcugucgacaaaauccagaaggugcugucgaauaagauacagauacagaggaacgagcaacuguaccuacugucaugacaaucuucuucauauucucuucaauuuuuauuuauuuuuuuucuuauuguucguguuuuauuuuuuuuuuuuaauuuccagauuagauuuaccu
......................uggaucuuguucUacagcacuc..............................................................................................................................................................................................................    2      1    JCK-1
......................uggaucuuguucgacagcacuA..............................................................................................................................................................................................................    9      1    JT-1
......................uggaucuuguucgacagcacuU..............................................................................................................................................................................................................    35     1    LT-3
......................uggaucuuguucgacagcGcuc..............................................................................................................................................................................................................    1      1    JCK-3
......................uNgaucuuguucgacagcacuc..............................................................................................................................................................................................................    1      1    LCK-3
......................uggaucuuguucgacagcacCc..............................................................................................................................................................................................................    2      1    JCK-2
......................uggaucuuguucgacGgcacuc..............................................................................................................................................................................................................    2      1    JCK-2
......................uggaucuuguucgacagcacuA..............................................................................................................................................................................................................    47     1    JCK-1
......................uggaucuuguucCacagcacuc..............................................................................................................................................................................................................    1      1    JCK-3
......................uggaucuuguucgacaAcacuc..............................................................................................................................................................................................................    1      1    LT-3
......................uAgaucuuguucgacagcacuc..............................................................................................................................................................................................................    1      1    LCK-3
......................uggaucuuguucgacagcacuc..............................................................................................................................................................................................................    188    0    JT-1
......................uggNucuuguucgacagcacuc..............................................................................................................................................................................................................    1      1    JCK-3
......................uggaucuuguucgacagcacCc..............................................................................................................................................................................................................    2      1    JCK-3
......................uUgaucuuguucgacagcacuc..............................................................................................................................................................................................................    2      1    JCK-3
......................uggaucuuguucgaUagcacuc..............................................................................................................................................................................................................    26     1    JCK-3
......................uAgaucuuguucgacagcacuc..............................................................................................................................................................................................................    2      1    LT-2
......................uggaucuCguucgacagcacuc..............................................................................................................................................................................................................    1      1    JCK-1
......................uggaucuuguucgacagcacuA..............................................................................................................................................................................................................    12     1    LT-2
......................uggaucuuguucgacagAacuc..............................................................................................................................................................................................................    1      1    JCK-1
......................uggaucuuguucgacagcacuA..............................................................................................................................................................................................................    67     1    JCK-3
......................uggaucuuguucgaUagcacuc..............................................................................................................................................................................................................    2      1    LT-1
......................uggaucuuguucgacagcacuc..............................................................................................................................................................................................................    955    0    LCK-3
......................uUgaucuuguucgacagcacuc..............................................................................................................................................................................................................    1      1    LCK-3
......................uggaucuuguucgacagcacuc..............................................................................................................................................................................................................    74     0    LCK-1
......................uggaucuuguucgacagcaAuc..............................................................................................................................................................................................................    1      1    JCK-1
......................uggaucuuguucgacagcacuA..............................................................................................................................................................................................................    24     1    LT-3
......................uggaucuuguucgacagcacuA..............................................................................................................................................................................................................    11     1    LCK-2
......................uAgaucuuguucgacagcacuc..............................................................................................................................................................................................................    4      1    JCK-2
......................ugAaucuuguucgacagcacuc..............................................................................................................................................................................................................    1      1    LT-1
......................uggaucuuguucCacagcacuc..............................................................................................................................................................................................................    5      1    JCK-2
......................uggGucuuguucgacagcacuc..............................................................................................................................................................................................................    1      1    LT-3
......................uNgaucuuguucgacagcacuc..............................................................................................................................................................................................................    9      1    JCK-3
......................uggaucuuguucgacagcacuA..............................................................................................................................................................................................................    19     1    LT-1
......................uAgaucuuguucgacagcacuc..............................................................................................................................................................................................................    1      1    LT-3
......................uggaucuuguucgacagcacuU..............................................................................................................................................................................................................    120    1    JCK-2
......................uUgaucuuguucgacagcacuc..............................................................................................................................................................................................................    1      1    JCK-1
......................uggaucuuguucgaAagcacuc..............................................................................................................................................................................................................    3      1    JCK-3
......................uggaucuuguucgacaAcacuc..............................................................................................................................................................................................................    1      1    JCK-1
......................uNgaucuuguucgacagcacuc..............................................................................................................................................................................................................    11     1    JCK-1
......................uggaucuuguucgacGgcacuc..............................................................................................................................................................................................................    2      1    LT-2
......................uggCucuuguucgacagcacuc..............................................................................................................................................................................................................    8      1    LT-2
......................uUgaucuuguucgacagcacuc..............................................................................................................................................................................................................    1      1    JCK-2
......................uggaucuuguucgacaAcacuc..............................................................................................................................................................................................................    4      1    JCK-2
......................uggaucuuguucgacagcacuc..............................................................................................................................................................................................................    2023   0    LT-1
......................uggaucuuguucgacagcCcuc..............................................................................................................................................................................................................    3      1    JCK-1
......................uggaucuuguucgacagcacuG..............................................................................................................................................................................................................    1      1    LT-3
......................uggaucuuguucgacagcacuc..............................................................................................................................................................................................................    14605  0    JCK-2
......................uggaucuuguucgacagcacuU..............................................................................................................................................................................................................    81     1    JCK-3
......................Cggaucuuguucgacagcacuc..............................................................................................................................................................................................................    3      1    JCK-3
......................uggaucuuguucgacagUacuc..............................................................................................................................................................................................................    5      1    JCK-2
......................uggaucuuguucgacagcGcuc..............................................................................................................................................................................................................    2      1    JCK-1
......................uggaucuuguucgacagcacuc..............................................................................................................................................................................................................    8479   0    JCK-1
......................uggaucuuguucgacagcaUuc..............................................................................................................................................................................................................    14     1    JCK-2
......................uggCucuuguucgacagcacuc..............................................................................................................................................................................................................    2      1    JT-1
......................uggaucuugCucgacagcacuc..............................................................................................................................................................................................................    5      1    JCK-2
......................uggaucuuguucgacagcCcuc..............................................................................................................................................................................................................    1      1    JCK-3
......................uggaucuuguucgacagcacucN.............................................................................................................................................................................................................    1      1    JCK-3
......................uggCucuuguucgacagcacucu.............................................................................................................................................................................................................    2      1    JCK-1
......................uggaucuuguucgacagcacucG.............................................................................................................................................................................................................    1      1    JCK-3
......................uggaucuuguucgacagcacucu.............................................................................................................................................................................................................    1104   0    JCK-3
......................uggaucuuguucgacagcacuUu.............................................................................................................................................................................................................    32     1    LCK-1
......................uggaucuuguuNgacagcacucu.............................................................................................................................................................................................................    3      1    JCK-1
......................uggaucuuguucgacagcacucA.............................................................................................................................................................................................................    52     1    JCK-3
......................uggaucuuguucgacagcacuAu.............................................................................................................................................................................................................    2      1    JCK-3
......................uNgaucuuguucgacagcacucu.............................................................................................................................................................................................................    1      1    JCK-2
......................uggCucuuguucgacagcacucu.............................................................................................................................................................................................................    3      1    JCK-2
......................uggaucuuguucgacagcacucu.............................................................................................................................................................................................................    27     0    LT-3
......................uggaucuuguucgacagcacuNu.............................................................................................................................................................................................................    2      1    JCK-1
......................uggNucuuguucgacagcacucu.............................................................................................................................................................................................................    2      1    JCK-1
```

```
                      Mature                                                Star
uccaucuuguucguuuacccucuggaucuuguucgacagcacucucuccuaucuuguuugacuacugaugaugagagugcugucgacaaaauccagaaggugcugucgaauaagauacagauacagaggaacgagcaacuguaccuacugucaugacaaucuucuucauauucucuucaauuuuuauuuauuuuuuuucuuauuguucguguuuuauuuuuuuuuuuuaauuuccagauuagauuuaccu
......................uggaucuuguucgaAgcacucu..............................................................................................................................................................................................................   1      1    LT-1
......................uggaucuuguucgacagcaUucu..............................................................................................................................................................................................................   1      1    JCK-1
......................uNgaucuuguucgacagcacucu..............................................................................................................................................................................................................   1      1    JCK-3
......................uggaucuuguucgacagcacucA..............................................................................................................................................................................................................   59     1    JCK-1
......................uggaucuuguucgCcagcacucu..............................................................................................................................................................................................................   1      1    JCK-1
......................uggaucuuguucgacagcacucu..............................................................................................................................................................................................................   19     0    JT-3
......................uggaucuuguucgCcagcacucu..............................................................................................................................................................................................................   2      1    LT-3
......................uggaucuuguucgacagcaNucu..............................................................................................................................................................................................................   1      1    JCK-1
......................uggaucuuguucgacagcacucG..............................................................................................................................................................................................................   4      1    JCK-2
......................uggaucuuguucgaUagcacucu..............................................................................................................................................................................................................   2      1    JCK-2
......................ugAaucuuguucgacagcacucu..............................................................................................................................................................................................................   1      1    JCK-3
......................uggaucuuguucgacagcacucu..............................................................................................................................................................................................................   1493   0    JCK-2
......................uggaucuuguucgacagcacuAu..............................................................................................................................................................................................................   1      1    LT-3
......................uggaucuuguucgacagcacuAu..............................................................................................................................................................................................................   1      1    JCK-1
......................ugAaucuuguucgacagcacucu..............................................................................................................................................................................................................   1      1    JCK-1
......................uggaucuuguucgacagcacucu..............................................................................................................................................................................................................   15     0    LT-2
......................uggaucuuguucgacagcacuUu..............................................................................................................................................................................................................   8      1    JT-2
......................uggaucuuguucgacagcacuUu..............................................................................................................................................................................................................   21     1    LT-1
......................uggaucuuguucgacagcacuGu..............................................................................................................................................................................................................   1      1    LT-3
......................uggaucuuguucgacagcacuUu..............................................................................................................................................................................................................   50     1    LCK-3
......................uggaucuuguucgacagUacucu..............................................................................................................................................................................................................   1      1    JCK-2
......................uggaucuuguucgCcagcacucu..............................................................................................................................................................................................................   2      1    JCK-2
......................ugUaucuuguucgacagcacucu..............................................................................................................................................................................................................   1      1    JCK-2
......................uggaucuuguucgacagcacucA..............................................................................................................................................................................................................   11     1    LT-3
......................uggaucuuguucgacagcacuGu..............................................................................................................................................................................................................   2      1    JCK-1
......................uggCucuuguucgacagcacucu..............................................................................................................................................................................................................   2      1    LT-2
......................uggaCcuuguucgacagcacucu..............................................................................................................................................................................................................   1      1    JCK-2
......................uggaucuuguucgacagcacuUu..............................................................................................................................................................................................................   20     1    LT-3
......................uggaucuuguucgacagcacucu..............................................................................................................................................................................................................   860    0    JCK-1
......................uggaucCuguucgacagcacucu..............................................................................................................................................................................................................   1      1    JCK-2
......................uAgaucuuguucgacagcacucu..............................................................................................................................................................................................................   1      1    JCK-3
......................uggaucuuguucgacagcacucG..............................................................................................................................................................................................................   1      1    LT-3
......................uggaucuuguucgacagcacucu..............................................................................................................................................................................................................   15     0    JT-2
......................uggaucuuguucgacagcacuUu..............................................................................................................................................................................................................   52     1    JCK-1
......................uggaucuuguucgacagcacuUu..............................................................................................................................................................................................................   22     1    LT-2
......................uggaucuuNuucgacagcacucu..............................................................................................................................................................................................................   1      1    JCK-3
......................uggaucuuguucgacagcacucA..............................................................................................................................................................................................................   14     1    LT-1
......................uggaucuuguucAacagcacucu..............................................................................................................................................................................................................   1      1    JCK-3
......................uggaucuuguucgacCgcacucu..............................................................................................................................................................................................................   1      1    JCK-3
......................uggaucuuguucgaUagcacucu..............................................................................................................................................................................................................   2      1    JCK-3
......................uggaucuuguucgacagcacuUu..............................................................................................................................................................................................................   52     1    JCK-3
......................uggaucuuguucgaUagcacucu..............................................................................................................................................................................................................   5      1    JCK-1
......................uggaucuuguucgacagcacuAu..............................................................................................................................................................................................................   2      1    JCK-2
......................uggaucuuguucgacagcacuUu..............................................................................................................................................................................................................   70     1    JCK-2
......................uggaucuuguucgacagcacucA..............................................................................................................................................................................................................   8      1    LT-2
......................uggaucuuguucgacagcacucu..............................................................................................................................................................................................................   15     0    LCK-3
......................uggaucuuguucgCcagcacucu..............................................................................................................................................................................................................   3      1    JCK-3
......................uNgaucuuguucgacagcacucu..............................................................................................................................................................................................................   2      1    JCK-1
......................uggaucuuguucgacagcacucA..............................................................................................................................................................................................................   18     1    LCK-3
......................uggCucuuguucgacagcacucu..............................................................................................................................................................................................................   3      1    JCK-3
......................uggaucuuguucgacagcacucA..............................................................................................................................................................................................................   64     1    JCK-2
......................uggaucuuguucgacagcacucG..............................................................................................................................................................................................................   5      1    LT-2
......................uggCucuuguucgacagcacucu..............................................................................................................................................................................................................   1      1    JT-1
......................uggaucuuguucgacagcacucu..............................................................................................................................................................................................................   4      0    JT-1
......................uggaucuuguucgacagcacuUu..............................................................................................................................................................................................................   5      1    JT-3
......................uggaucuuguucgacagcacuUu..............................................................................................................................................................................................................   17     1    LCK-2
......................uggaucuuguucgacagcacuAu..............................................................................................................................................................................................................   3      1    LT-1
......................uggaucuuguucgacagcacCcu..............................................................................................................................................................................................................   1      1    JCK-2
......................uggaucuuguucgacagcacucu..............................................................................................................................................................................................................   14     0    LT-1
......................uggaucuuCuucgacagcacucu..............................................................................................................................................................................................................   1      1    JCK-3
......................Cggaucuuguucgacagcacucu..............................................................................................................................................................................................................   1      1    JCK-1
......................uggaucuuguucgacagcacucuc.............................................................................................................................................................................................................   11     0    JCK-1
......................uggaucuuguucgacagcacucuc.............................................................................................................................................................................................................   6      0    LT-2
......................uggaucuuguucgacagcacucuU.............................................................................................................................................................................................................   1      1    LT-1
......................uggaucuuguucgacagcacucuA.............................................................................................................................................................................................................   1      1    LCK-3
......................uggaucuuguucgacagcacucuc.............................................................................................................................................................................................................   7      0    LT-1
......................uggaucuuguucgacagcacucuA.............................................................................................................................................................................................................   1      1    LT-2
......................uggaucuuguucgacagcacucuc.............................................................................................................................................................................................................   10     0    JCK-2
......................uggaucuuguucgacagcacucuU.............................................................................................................................................................................................................   12     1    JCK-1
......................uggaucuuguucgacagcacucuU.............................................................................................................................................................................................................   10     1    JCK-3
```

Mature Star

uccaucuuguucguuuacccucuggaucuuguucgacagcacucucuccuaucuuguuugacuacugaugaugagagugcugucgacaaaauccagaaggugcugucgaauaagauacagauacagaggaacgagcaacuguaccuacugucaugacaaucuucuucauauucucuucaauuuuuauuuauuuuuuuucuuauuguucguguuuuauuuuuuuuuuuuaauuuccagauuagauuuaccu

......................uggaucuuguucgacagcacucuc	5	0	LT-3
......................uggaucuuguucgacagcacucuU	13	1	JCK-2
......................uggaucuuguucgacagcacucuA	17	1	JCK-1
......................uggaucuuguucgacagcacuUuc	2	1	JCK-3
......................uggaucuuguucgacagcacucuc	10	0	JCK-3
......................uggaucuuguucgacagcacucuU	3	1	LT-2
......................uggaucuuguucgacagcacuUuc	5	1	LCK-3
......................uggaucuuguucgacagcacucuA	3	1	LT-3
......................uggaucuuguucgacagcacucuA	22	1	JCK-3
......................uggaucuuguucgacagcacucuA	31	1	JCK-2
......................uggaucuuguucgacagcacuUuc	6	1	JCK-2
......................uggaucuuguucgacagcacucuG	1	1	JCK-1
......................uggaucuuguuNgacagcacucuc	1	1	JCK-1
......................uggaucuuguucgacagcacuAuc	1	1	JCK-2
......................uggaucuuguucgacagcacuUuc	2	1	JCK-1
......................uggaucuuguucgacagcacuGuc	3	1	JCK-2
......................uggaucuuguucgacagcacucucu	1	0	JCK-2
......................uggaucuuguucgacagcacucuUu	3	1	LT-1
......................uggaucuuguucgacagcacucucu	10	0	LCK-3
......................uggaucuuguucgacagcacucuUu	2	1	LT-3
......................uggaucuuguucgacagcacucCcu	1	1	LT-1
......................uggaucuuguucgacagcacucucu	2	0	LT-3
......................uggaucuuguucgacagcacucucA	1	1	LT-3
......................uggaucuuguucgacagcacucucu	1	0	LT-1
......................uggaucuuguucgacagcacucuAuc	1	1	JCK-2
......................uggaucuuguucgacagcacucucCc	1	1	LT-3
......................uggaucuuguucgacagcacucuAuc	1	1	JCK-3
......................uggaucuuguucgacagcacucuAuc	1	1	JCK-1
.......................ggaucuuguucgacagca	5	0	LT-2
.......................ggaucuuguucgacagca	2	0	JCK-1
.......................ggaucuuguucgacagca	10	0	JT-1
.......................ggaucuuguucgacagcacu	1	0	LT-2
.......................ggaucuuguucgacagcacu	2	0	JCK-1
.......................Ugaucuuguucgacagcacu	1	1	LT-1
.......................ggaucuuguucgacagcacu	5	0	JCK-3
.......................ggaucuuguucgacagcacu	1	0	LT-1
.......................ggaucuuguucgacagcacu	1	0	JCK-2
.......................ggaucuuguucgacagcacuc	2	0	JCK-3
.......................ggaucuuguucgacagcacuc	1	0	LT-1
.......................ggaucuuguucgacagcacuc	3	0	JCK-2
.......................ggaucuuguucgacagcacucu	1	0	JCK-2
.......................ggaucuuguucgacagcacucu	2	0	JCK-3
........................gaucuuguucgacagcacu	2	0	JCK-3
........................gaucuuguucgacagcacu	2	0	JCK-1
........................gaucuuguucgacagcacucu	1	0	JCK-3
........................gaucuuguucgacagcacucuc	2	0	JCK-2
........................gaucuuguucgacagcacucuc	3	0	JCK-3
.........................aucuuguucgacagcacu	1	0	LT-3
.........................aucuuguucgacagcacuc	3	0	JCK-3
.........................aucuuguucgacagcacuc	1	0	LT-3
.........................aucuuguucgacagcacuc	2	0	JCK-2
.........................aucuuguucgacagcacuc	2	0	JCK-1
.........................aucuuguucgacagcacucu	1	0	LT-3
.........................aucuuguucgacagcacucu	1	0	LT-1
.........................aucuuguucgacagcacucu	2	0	JCK-2
.........................aucuuguucgacagcacuUu	2	1	JCK-3
.........................aucuuguucgacagcacucuc	2	0	LT-1
.........................aucuuguucgacagcacucuc	1	0	JCK-2
.........................aucuuguucgacagcacucuc	2	0	LT-3
.........................aucuuguucgacagcacucuc	2	0	JCK-1
.........................aucuuguucgaNagcacucuc	1	1	JCK-1
.........................aucuuguucgacagcacucuc	1	0	JCK-3
.........................aucuuguucgacagcacucuUu	1	1	JCK-2
.........................aucuuguucgacagcacucuUu	1	1	JCK-3
.........................aucuuguucgacagcacucucu	4	0	JCK-2
.........................Cucuuguucgacagcacucucu	1	1	JCK-1
.........................aNcuuguucgacagcacucucu	1	1	JCK-3
.........................aucuuguucgacagcacucucu	3	0	LT-3
.........................aucuuguucgacagcacucucu	7	0	JCK-3
.........................aucuuguucgacagcacucucu	6	0	LCK-3

Mature **Star**

uccaucuuguucguuuacccucuggaucuuguucgacagcacucucuccuaucuuguuugacuacugaugaugagagugcugucgacaaaauccagaaggugcugucgaauaagauacagauacagaggaacgagcaacuguaccuacugucaugacaaucuucuucauauucucuucaauuuuuauuuauuuuuuuucuuauuguucguguuuuauuuuuuuuuuuaauuuccagauuagauuuaccu

aucuuguucgacagcacucucu	4	0	JCK-1
aucuuguucgacagcacucucu	1	0	LT-1
aucuuguucgacagcacucucuU	1	1	JCK-1
ucuuguucgacagcacuc	1	0	LT-1
ucuuguucgacagcacuc	2	0	LT-3
ucuuguucgacagcacucu	1	0	LT-3
ucuuguucgacagcacucuc	1	0	JCK-3
ucuuguucgacagcacucucu	1	0	LT-3
cacucucuccuaucuuguuu	1	0	LT-3
ucuccuaucuuguuugacuacugauga	1	0	JCK-3
ucuccuaucuuguuugacuacugauga	1	0	JCK-2
uaucuuguuugacuacugaugaugaga	1	0	LT-1
uaucuuguuugacuacugaugaugagagu	2	0	LT-1
ucuuguuugacuacugaugaugagagug	1	0	JT-1
cuuguuugacuacugaug	1	0	LT-3
uuguuugacuacugaugaugaga	5	0	LCK-3
uuugacuacugaugaugaga	1	0	JCK-2
uuugacuacugaugaugaga	2	0	JCK-3
uugacuacugaugaugaga	1	0	LT-3
cugaugaugagagugcuguc	3	0	JCK-1
cugaugaugagagugcugucgacaaGauc	2	1	JT-2
ugaugagagugcugucgacaaGauc	2	1	JCK-2
ugaugagagugcugucgacaaGauc	1	1	JCK-1
ugaugagagugcugucgacaaGauc	1	1	JCK-3
ugagagugcugucgacaaGauc	1	1	JCK-1
ugagagugcugucgacaaaauc	6	0	LCK-3
ugagagugcugucgacaaGauc	1	1	JCK-2
ugagagugcugucgacaaaauc	1	0	LT-2
ugagagugcugucgacaaaaucc	1	0	LT-3
gagagugcugucgacaaGauc	2	1	JCK-1
agagugcugucgacaaGau	1	1	JCK-1
agagugcugucgacaaaau	4	0	LCK-3
agagugcugucgacaaaau	2	0	LT-1
agagugcugucgacaaGau	2	1	JCK-2
agagugcugucgacaaGau	1	1	JCK-3
agagugcugucgacaaaau	1	0	JCK-2
agagugcugucgacaaGau	22	1	JT-1
agagugcugucgacaaaauc	4	0	LT-3
agagugcugucgacaaaauc	6	0	LT-2
agagugcugucgacaaaauc	1	0	JCK-2
agagugcugucgacaaGauc	10	1	JT-1
agagugcugucgacaaGauc	70	1	JCK-2
agagugcugucgacaaGauc	55	1	JCK-3
agagugcugucgacaaGauc	51	1	JCK-1
Cgagugcugucgacaaaauc	1	1	LT-2
agagugcugucgacaaaauc	1	0	LT-1
agagugcugucgacaaaaucc	5	0	LT-1
agagugcugucgacaaGaucc	9	1	JT-1
agagugcugucgacaaGaucc	31	1	JCK-2
agagugcugucgacaaaaucc	3	0	JCK-3
agagugcugucgacaaaaucc	3	0	JCK-2
agagugcugucgacaaaaucc	8	0	LCK-3
agagugcugucgacaaGaucc	24	1	JCK-3
agagugcugucgacaaaaucc	3	0	JCK-1
agagugcugucgacaaaaucc	3	0	LT-3
agagugcugucgacaaGaucc	24	1	JCK-1
agagugcugucgacaaaaucca	2	0	LT-3
agagugcugucgacaaGaucca	2	1	JT-3
agagugcugucgacaaaaucca	3	0	LT-2
agagugcugucgacaaGaucca	1	1	JCK-1
agagugcugucgacaaGaucca	2	1	JCK-2
agagugcugucgacaaaaucca	1	0	LT-1
agagugcugucgacaaGaucca	2	1	JT-2
agagugcugucgacaaaaucca	2	0	JCK-2
agagugcugucgacaaGauccag	2	1	JCK-3
agagugcugucgacaaaauccaA	2	1	JCK-2
agagugcugucgacaaaauccag	1	0	JCK-1
agagugcugucgacaaaauccag	1	0	LT-3
agagugcugucgacaaaauccaga	18	0	JCK-3
agagugcugucgacaaGauccaga	36	1	JCK-2

Mature **Star**

uccaucuuguucguuuacccucuggaucuuguucgacagcacucucuccuaucuuguuugacuacugaugaugagagugcugucgacaaaauccagaaggugcugucgaauaagauacagauacagaggaacgagcaacuguaccuacugucaugacaaucuucuucauauucucuucaauuuuuauuuauuuuuuuucuuauuguucguguuuuauuuuuuuuuuuuaauuuccagauuagauuuaccu

agagugcugucgacaaaauccaga	4	0	LCK-3
agagugcugucgacaaGauccaga	19	1	JCK-1
agagugcugucgacaaaauccaga	9	0	LT-2
agagugcugucgacaaGauccaga	23	1	JCK-3
agagugcugucgacaaaauccaga	7	0	LT-3
agagugcugucgacaaaauccaga	15	0	JCK-2
agagugcugucgacaaaauccaga	35	0	LCK-2
agagugcugucgacaaaauccaga	10	0	LT-1
agagugcugucgacaaaauccaga	5	0	JCK-1
agagugcugucgacaaaauccagG	1	1	LCK-1
agagugcugucgacaaaaucNaga	1	1	JCK-1
agagugcugucgacaaaauccaga	43	0	LCK-1
agagugcugucgacaaaauccagaa	2	0	JCK-1
agagugcugucgacaaaauccagaa	1	0	JCK-3
agagugcugucgacaaaauccagaa	1	0	JCK-2
agagugcugucgacaaaauccagaa	2	0	LT-3
gagugcugucgacaaGaucc	2	1	JCK-1
gagugcugucgacaaGaucc	10	1	JCK-3
gagugcugucgacaaaaucc	1	0	LT-3
gagugcugucgacaaGaucc	9	1	JCK-2
gagugcugucgacaaGaucc	5	1	JT-2
gagugcugucgacaaaaucc	1	0	JCK-3
gagugcugucgacaaaaucc	2	0	JCK-2
gagugcugucgacaaGaucc	8	1	JT-3
gagugcugucgacaaGaucca	15	1	JCK-2
gagugcugucgacaaGaucca	8	1	JCK-1
gagugcugucgacaaGaucca	8	1	JCK-3
gagugcugucgacaaaaucca	1	0	JCK-2
gagugcugucgacaaaauccag	1	0	JCK-2
gagugcugucgacaaaauccaga	11	0	JCK-2
gagugcugucgacaaaauccaga	7	0	JCK-1
gagugcugucgacaaGauccaga	44	1	JCK-3
gagugcugucgacaaGauccaga	72	1	JCK-2
gagugcugucgacaaaauccaga	5	0	LT-3
gagugcugucgacaaaauccaga	12	0	JCK-3
gagugcugucgacaaGauccaga	35	1	JCK-1
gagugcugucgacaaaauccaga	1	0	LT-1
gagugcugucgacaaaauccaga	4	0	LT-2
agugcugucgacaaaauc	3	0	LCK-3
agugcugucgacaaGaucc	3	1	JCK-3
agugcugucgacaaaaucU	2	1	LCK-3
agugcugucgacaaGaucc	7	1	JCK-2
agugcugucgacaaGaucca	9	1	JCK-2
agugcugucgacaaGaucca	2	1	JCK-1
agugcugucgacaaGaucca	4	1	JCK-3
agugcugucgacaaGauccag	2	1	JCK-2
agugcugucgacaaGauccag	5	1	JCK-1
agugcugucgacaaGauccag	4	1	JCK-3
agugcugucgacaaaauccaga	3	0	JCK-2
agugcugucgacaaaauccaga	2	0	JCK-3
agugcugucgacaaaauccaga	1	0	LT-3
agugcugucgacaaGauccaga	5	1	JCK-1
agugcugucgacaaGauccaga	1	1	JCK-3
agugcugucgacaaGauccaga	8	1	JCK-2
agugcugucgacaaGauccagaa	1	1	JCK-2
agugcugucgacaaaauccagaag	1	0	LT-1
gugcugucgacaaaaucc	5	0	LT-3
gugcugucgacaaaaucc	3	0	LCK-3
gugcugucgacaaaaucc	2	0	LT-2
gugcugucgacaaGaucc	16	1	JCK-1
gugcugucgacaaGaucc	3	1	JT-2
gugcugucgacaaGaucc	42	1	JCK-2
gugcugucgacaaaaucc	3	0	LT-1
gugcugucgacaaGaucc	5	1	JT-3
gugcugucgacaaGaucc	18	1	JCK-3
gugcugucgacaaaaucc	2	0	JCK-3
gugcugucgacaaaaucca	7	0	LT-2
gugcugucgacaaaaucca	1	0	JCK-2
gugcugucgacaaaaucca	4	0	JT-2
gugcugucgacaaGaucca	11	1	JCK-1

```
                      Mature                                                  Star
uccaucuuguucguuuacccucuggaucuuguucgacagcacucucuccuaucuuguuugacuacugaugaugagagugcugucgacaaaauccagaaggugcugucgaauaagauacagauacagaggaacgagcaacuguaccuacugucaugacaaucuucuucauauucucuucaauuuuuauuuauuuuuuuucuuauuguucguguuuuauuuuuuuuuuuaauuuccagauuagauuuaccu
............................................................................gugcugucgacaaaaucca..........................................................................................................................................................   1     0   JCK-1
............................................................................gugcugucgacaaaaucca..........................................................................................................................................................   4     0   LCK-3
............................................................................gugcugucgacaaaaucca..........................................................................................................................................................   4     0   LT-3
............................................................................gugcugucgacaaaaucca..........................................................................................................................................................   3     0   JCK-3
............................................................................gugcugucgacaaaaucca..........................................................................................................................................................   3     0   JT-3
............................................................................gugcugucgacaaaaucca..........................................................................................................................................................   9     0   JT-1
............................................................................gugcugucgacaaGaucca..........................................................................................................................................................   27    1   JCK-2
............................................................................gugcugucgacaaGaucca..........................................................................................................................................................   7     1   JT-2
............................................................................gugcugucgacaaaaucca..........................................................................................................................................................   1     0   LT-1
............................................................................gugcugucgacaaGaucca..........................................................................................................................................................   9     1   JCK-3
............................................................................gugcugucgacaaGaucca..........................................................................................................................................................   8     1   JT-3
............................................................................gugcugucgacaaGaucca..........................................................................................................................................................   7     1   JT-1
............................................................................gugcugucgacaaGauccag.........................................................................................................................................................   11    1   JCK-3
............................................................................gugcugucgacaaGauccag.........................................................................................................................................................   9     1   JT-2
............................................................................gugcugucgacaaGauccag.........................................................................................................................................................   7     1   JCK-1
............................................................................gugcugucgacaaGauccag.........................................................................................................................................................   9     1   JT-3
............................................................................gugcugucgacaaGauccag.........................................................................................................................................................   32    1   JCK-2
............................................................................gugcugucgacaaGauccaga........................................................................................................................................................   18    1   JT-2
............................................................................gugcugucgacaaGauccaga........................................................................................................................................................   910   1   JCK-3
............................................................................gCgcugucgacaaaauccaga........................................................................................................................................................   1     1   JCK-1
............................................................................gCgcugucgacaaaauccaga........................................................................................................................................................   1     1   JCK-3
............................................................................gugcugucgacaaGauccaga........................................................................................................................................................   19    1   JT-1
............................................................................gugcugucgacaaaauccaga........................................................................................................................................................   12    0   LT-3
............................................................................gugcugucgacaaaaucUaga........................................................................................................................................................   2     1   JCK-2
............................................................................gugcugucgacaaaauccaAa........................................................................................................................................................   1     1   JCK-1
............................................................................gugcugucgacaaaauccaNa........................................................................................................................................................   1     1   JCK-1
............................................................................gugcugucgacaaGauccaga........................................................................................................................................................   712   1   JCK-1
............................................................................gugcugucgacaaGauccaga........................................................................................................................................................   22    1   JT-3
............................................................................gugcugucgacaaaaucUaga........................................................................................................................................................   1     1   JCK-3
............................................................................gugcugucgacaaaauccaga........................................................................................................................................................   27    0   JCK-2
............................................................................gugcugucgacaaaauccaga........................................................................................................................................................   18    0   LT-1
............................................................................gugcugucgacaaaauccaga........................................................................................................................................................   20    0   JCK-3
............................................................................gCgcugucgacaaaauccaga........................................................................................................................................................   1     1   JCK-2
............................................................................gugcugucgacaaaauccaAa........................................................................................................................................................   2     1   JCK-2
............................................................................gugcugucgacaaaauccaga........................................................................................................................................................   23    0   JCK-1
............................................................................gugcugucgacaaGauccaga........................................................................................................................................................   1129  1   JCK-2
............................................................................gugcugucgacaUaauccaga........................................................................................................................................................   1     1   JCK-2
............................................................................gugcugucgacaaaauccaga........................................................................................................................................................   11    0   LT-2
............................................................................gugcugucgacaaGauccagaa.......................................................................................................................................................   2     1   JCK-3
............................................................................gugcugucgacaaGauccagaa.......................................................................................................................................................   7     1   JCK-2
............................................................................gugcugucgacaaGauccagaa.......................................................................................................................................................   6     1   JCK-1
............................................................................gugcugucgacaaaauccagaa.......................................................................................................................................................   1     0   LT-1
............................................................................Augcugucgacaaaauccagaa.......................................................................................................................................................   1     1   LT-1
............................................................................gugcugucgacaaaauccagaagg.....................................................................................................................................................   2     0   LT-1
.............................................................................ugcugucgacaaGauccag.........................................................................................................................................................   2     1   JCK-1
.............................................................................ugcugucgacaaGauccag.........................................................................................................................................................   2     1   JCK-2
.............................................................................ugcugucgacaaGauccag.........................................................................................................................................................   2     1   JCK-3
.............................................................................ugcugucgacaaaauccaga........................................................................................................................................................   1     0   LT-3
.............................................................................ugcugucgacaaGauccaga........................................................................................................................................................   125   1   JCK-2
.............................................................................ugcugucgacaaGauccaga........................................................................................................................................................   20    1   JT-3
.............................................................................ugcugucgacaaaauccaga........................................................................................................................................................   3     0   LT-1
.............................................................................ugcugucgacaaaauccaga........................................................................................................................................................   2     0   LCK-3
.............................................................................ugcugucgacaaGauccaga........................................................................................................................................................   105   1   JCK-3
.............................................................................ugcugucgacaaGauccaga........................................................................................................................................................   22    1   JT-1
.............................................................................ugcugucgacaaGauccaga........................................................................................................................................................   13    1   JT-2
.............................................................................ugcugucgacaaGauccaga........................................................................................................................................................   79    1   JCK-1
.............................................................................ugcugucgacaaGauccagaa.......................................................................................................................................................   2     1   JCK-1
.............................................................................ugcugucgacaaGauccagaa.......................................................................................................................................................   1     1   JCK-3
.............................................................................ugcugucgacaaGauccagaaggu....................................................................................................................................................   1     1   JCK-2
..............................................................................gcugucgacaaGauccagaag......................................................................................................................................................   3     1   JT-2
..............................................................................gcugucgacaaGauccagaag......................................................................................................................................................   1     1   JCK-2
..............................................................................gcugucgacaaGauccagaag......................................................................................................................................................   3     1   JCK-3
..............................................................................gcugucgacaaGauccagaag......................................................................................................................................................   3     1   JCK-1
..............................................................................gcugucgacaaGauccagaag......................................................................................................................................................   4     1   JT-3
...............................................................................cugucgacaaGauccaga........................................................................................................................................................   1     1   JCK-2
...............................................................................cugucgacaaaauccaUa........................................................................................................................................................   1     1   LT-3
...............................................................................cugucgacaaGauccaga........................................................................................................................................................   1     1   JCK-1
...............................................................................cugucgacaaGauccagaa.......................................................................................................................................................   1     1   JCK-3
...............................................................................cugucgacaaGauccagaa.......................................................................................................................................................   2     1   JCK-1
...............................................................................cugucgacaaGauccagaa.......................................................................................................................................................   2     1   JCK-2
```

```
                      Mature                                                Star
uccaucuuguucguuuacccucuggaucuuguucgacagcacucucuccuaucuuguuugacuacugaugaugagagugcugucgacaaaauccagaaggugcugucgaauaagauacagauacagaggaacgagcaacuguaccuacugucaugacaaucuucuucauauucucuucaauuuuuauuuauuuuuuuucuuauuguucguguuuuauuuuuuuuuuuaauuuccagauuagauuuaccu
...............................................................................cugucgacaaGauccagaag......................................................................................................................................................   1    1    JCK-1
...............................................................................cugucgacaaGauccagaag......................................................................................................................................................   5    1    JCK-3
...............................................................................cugucgacaaGauccagaag......................................................................................................................................................   4    1    JCK-2
...............................................................................cugucgacaaGauccagaagg.....................................................................................................................................................   3    1    JCK-3
...............................................................................cugucgacaaGauccagaagg.....................................................................................................................................................   4    1    JCK-2
...............................................................................cugucgacaaGauccagaagg.....................................................................................................................................................   5    1    JCK-1
...............................................................................cugucgacaaGauccagaaggugcu.................................................................................................................................................   1    1    JCK-2
................................................................................ugucgacaaGauccagaagg.....................................................................................................................................................   1    1    JCK-1
................................................................................ugucgacaaGauccagaaggu....................................................................................................................................................   6    1    JCK-1
................................................................................ugucgacaaGauccagaaggu....................................................................................................................................................   17   1    JCK-3
................................................................................ugucgacaaGauccagaaggu....................................................................................................................................................   11   1    JCK-2
..................................................................................ucgacaaGauccagaaggugc..................................................................................................................................................   3    1    JCK-3
..................................................................................ucgacaaGauccagaaggugc..................................................................................................................................................   2    1    JCK-1
..................................................................................ucgacaaGauccagaaggugc..................................................................................................................................................   1    1    JCK-2
..................................................................................ucgacaaaauccagaaggugc..................................................................................................................................................   2    0    LT-1
.....................................................................................acaaGauccagaaggugcugucga............................................................................................................................................   2    1    JCK-1
.....................................................................................acaaGauccagaaggugcugucga............................................................................................................................................   2    1    JCK-3
.....................................................................................acaaGauccagaaggugcugucga............................................................................................................................................   1    1    JCK-2
.....................................................................................acaaGauccagaaggugcugucgaauaaga......................................................................................................................................   1    1    JCK-2
.....................................................................................acaaGauccagaaggugcugucgaauaaga......................................................................................................................................   1    1    JCK-3
.........................................................................................aauccagaaggugcugucgaauU.........................................................................................................................................   1    1    LT-3
.............................................................................................cagaaggugcugucgaauaag.......................................................................................................................................   1    0    LT-3
.............................................................................................cagaaggugcugucgaauaagaua....................................................................................................................................   8    0    JCK-1
.................................................................................................aggugcugucgaauaagaua....................................................................................................................................   2    0    LT-2
.................................................................................................aggugcugucgaauaagaua....................................................................................................................................   2    0    JCK-1
.................................................................................................aggugcugucgaauaagaua....................................................................................................................................   1    0    JCK-2
.................................................................................................aggugcugucgaauaagauaca..................................................................................................................................   1    0    LT-1
...................................................................................................gugcugucgaaCaagaua....................................................................................................................................   1    1    JCK-2
...................................................................................................gugcugucgaaCaagaua....................................................................................................................................   2    1    LCK-3
..................................................................................................................................acgagcaacuguaccuacugu..................................................................................................   1    0    JCK-1
..................................................................................................................................acgagcaacuguaccuacugu..................................................................................................   2    0    JCK-2
```
